# The Application of Untargeted Metabolomic Approaches for the Search of Common Bioavailable Metabolites in Human Plasma Samples from *Lippia citriodora* and *Olea europaea* Extracts

**DOI:** 10.1021/acs.jafc.4c05325

**Published:** 2024-10-22

**Authors:** María del Carmen Villegas-Aguilar, María de la Luz Cádiz-Gurrea, Noelia Sánchez-Marzo, Enrique Barrajón-Catalán, David Arráez-Román, Álvaro Fernández-Ochoa, Antonio Segura-Carretero

**Affiliations:** † Department of Analytical Chemistry, University of Granada, 18071 Granada, Spain; ‡ Institute of Research, Development and Innovation in Biotechnology of Elche (IDiBE) and Molecular and Cell Biology Institute (IBMC), Miguel Hernández University (UMH), 03202 Elche, Spain

**Keywords:** Lippia citriodora, Olea europaea, metabolomics, untargeted, bioavailability, metabolism, mass spectrometry, liquid chromatography, bioactive compounds, phenolic compounds

## Abstract

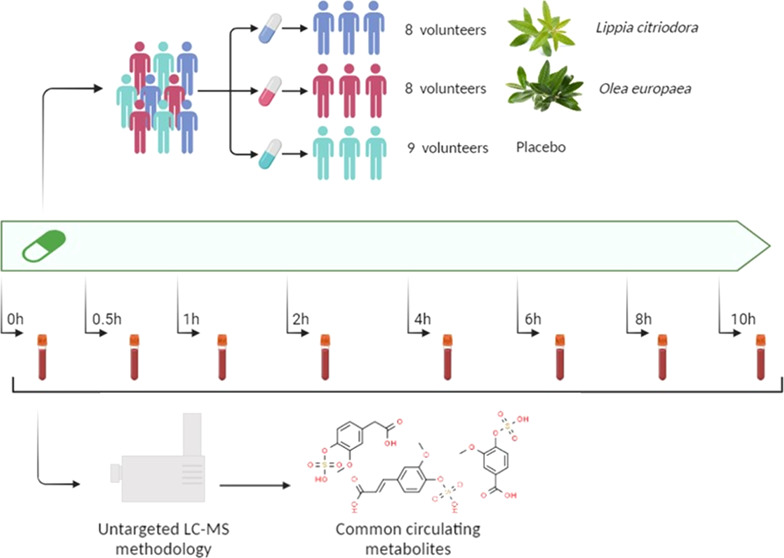

*Lippia citriodora* and *Olea europaea* are known for their shared common bioactivities. Although both matrices are rich in similar families of bioactive compounds, their specific phytochemical compounds are mostly different. Since these compounds can be metabolized in the organism, this study hypothesized that common bioavailable metabolites may contribute to their similar bioactive effects. To test this, an acute double-blind intervention study in humans was conducted with blood samples collected at multiple time points. Using an untargeted metabolomic approach based on HPLC-ESI-QTOF-MS, 66 circulating metabolites were detected, including 9 common to both extracts, such as homovanillic acid sulfate and glucuronide derivates, hydroxytyrosol sulfate, etc. These common metabolites displayed significantly different *T*_max_ values depending on the source, suggesting distinct metabolization pathways for each extract. The study highlights how shared bioavailable metabolites may underlie similar bioactivities observed between these two plant sources.

## 1 Introduction

Two plant matrices whose phenolic composition has been extensively studied are *Lippia citriodora* (LC) and *Olea europaea* (OE). Both matrices have a common bioactivity, since both species have potent antioxidant,^1,2^ anti-inflammatory,^3^ antimicrobial,^4^ and antitumor properties.^5^ However, although both matrices share similar bioactive properties, their phenolic compositions differ significantly. LC is characterized by a high content of glycosylated phenylpropanoids and iridoids and OE by the presence of secoiridoids.^6^

The bioactive compounds in both matrices exhibit strong biological activity. The bioactivity of these compounds has traditionally been investigated first through *in vitro* studies, followed by *in vivo* assays. However, these compounds may undergo metabolic transformations before reaching their therapeutic targets.^7^ In this regard, ingested phenolic compounds may first be hydrolyzed by gastric fluids in the stomach and subsequently metabolized by enzymes in the intestinal cells or by the colonic microbiota.^8^ Common transformations in such compounds include the removal of sugar moieties, resulting in the formation of aglycones from the original molecules.^9^ Additionally, these compounds may undergo phase I and II metabolic biotransformations in the liver. Major phase I reactions include oxidation and reduction, while phase II processes involve methylation, sulfation, or glucuronidation.^9^ In the literature, there are few studies that have investigated the bioavailability and metabolism of compounds present in LC and OE. These studies have been conducted in rats for LC^10^ and in both rats and humans for OE.^10,11^ Examples of phase II metabolites derived from compounds detected in LC and OE include hydroxytyrosol sulfate and hydroxytyrosol glucuronide.^12^ However, there is a lack of research specifically focusing on the search for bioactive compounds from both plant sources conducted in either animals or humans.

The methodologies commonly used in studies of bioavailability and metabolism rely on targeted or semitargeted metabolomic approaches.^13^ This type of methodology focuses on the analysis of predefined metabolites from the original composition of the extracts or potential predictions of metabolites derived through known metabolization mechanisms.^14^ While these approaches allow for the detection and quantification of known metabolites, they are limited in their ability to discover unexpected bioavailable metabolites.^15^ To overcome this limitation, untargeted metabolomics strategies cover the entire range of detected compounds in the analysis without a predefined list of candidates. This type of approach has hardly been used in bioavailability and metabolism studies. Although quantification is not possible using untargeted approaches, these methodologies have the great advantage of discovering new or unexpected metabolites.^13,16,17^

Based on this context, we hypothesize that common bioavailable metabolites from OE and LC may be responsible for their similar bioactive effects. Given that the phytochemical compositions are mostly different, the potential common bioavailable compounds may be unexpected, and therefore, the untargeted metabolomic approaches have great potential to resolve the hypothesis. Identifying these metabolites is crucial, as they can be distributed through the bloodstream to target tissues where they may exert beneficial biological effects. Then, the present study aimed to detect circulating blood metabolites from LC and OE extracts using an untargeted metabolomics approach applied in an acute nutritional intervention assay in humans.

## 2 Materials and Methods

### 2.1 Chemicals

All solvents used for metabolite analysis were of analytical reagent grade and were used as received. Formic acid was purchased from Fluka, Sigma-Aldrich (Steinheim, Germany). Water was purified using a Milli-Q system from Millipore (Bedford, MA). Ethanol and methanol (Fisher Scientific Madrid, Spain) for plasma treatment were of LC-MS grade. The chemical standards oleuropein (≥98%), hydroxytyrosol (≥98%), and verbascoside (≥97%) were purchased from Sigma-Aldrich (St. Louis, MO). Hydroxytyrosol glucuronide (≥98%), homovanilic acid sulfate sodium salt (≥96%), and vanillic acid 4-sulfate sodium salt (≥97%) were purchased from TRC-Canada (Toronto, Ontario, Canada).

### 2.2 OE and LC Extracts

The bioactive extracts were selected based on their composition and bioactive potential, the results of which have been previously published.^18^ In summary, NATAC Biotech S.L. (Cáceres, Spain) supplied preindustrial extracts derived from two different plant matrices. Both extracts were obtained through a solid–liquid extraction process using a mixture of ethanol and water in a ratio of 80:20 (v:v) for a duration of 2 h, maintaining a solvent-to-plant ratio of 20:1. The extraction temperatures were set to 45 °C for the OE and 55 °C for the LC, respectively. After extraction, the extracts were vacuum-dried, stored at room temperature, and protected from light until the encapsulation process. Considering that most commercial supplements based on these plant matrices range in doses between 250 and 600 mg, the extract was encapsulated in a 500 mg dosage form. The OE and LC extracts were previously qualitatively characterized by HPLC-ESI-QTOF-MS.^18^ The main compounds were quantified in the current study as described in Section 2.6 for a better discussion of the biological results.

### 2.3 Subjects and Study Design

The study protocol was conducted in accordance with the ethical standards set forth in the Declaration of Helsinki and received approval from the Ethics Committee of Miguel Hernández University of Elche and the General University Hospital of Elche (Alicante, Spain), with reference number PI 57/2019. A total of 25 healthy individuals participated, with an average age of 27 ± 9 years and a mean body mass index (BMI) of 23 ± 3 kg/m^2^. None of the participants were taking any medications or nutritional supplements nor did they have any chronic diseases or gastrointestinal issues. The sample size was established based on findings from previous bioavailability research.^14^ All participants provided informed consent before participating in the study.

The intervention study was carried out at Miguel Hernández University. Following an overnight fast, the volunteers were categorized into the subsequent groups: a subgroup to evaluate the LC leaf extract (*n* = 8), another for the OE leaf extract (*n* = 8), and the placebo subgroup (*n* = 9). This was a double-blind study, in which sample collection and treatment were carried out uniformly across all three subgroups.

A polyphenol-free breakfast was offered 30 min after consumption of the encapsulation; 2, 4, and 9 h later (2.5, 4.5, and 9.5 h after ingestion of the encapsulated extracts, respectively), a polyphenol-free snack and a lunch were also offered to the volunteers. Water was provided ad libitum. Prior to the ingestion of the capsule, a nurse inserted a cannula into the ulnar vein of each volunteer’s nondominant arm, and blood samples were drawn into EDTA-coated tubes at baseline (*t* = 0). Blood samples were then collected at 0.5, 1, 2, 4, 6, 8, and 10 h after the consumption of the 500 mg capsule. Plasma was separated through centrifugation (10 min at 3000 rpm and 4 °C) and stored at −80 °C until further analysis.

### 2.4 HPLC-ESI-QTOF-MS Plasma Analysis

Plasma samples were initially treated using a mixture of methanol and ethanol (50:50; v:v) to remove proteins, following previously reported protocols.^19^ During the sample treatment, pooled quality controls (pooled-QC) were prepared by combining equal aliquots from each sample. These QC samples were processed by using the same protocols as the experimental biological samples.

Once the biological samples were treated, they were analyzed on an Agilent 1260 HPLC instrument (Agilent Technologies, Palo Alto, CA) coupled to an Agilent 6540 Ultra High Definition (UHD) Accurate Mass QTOF equipped with a dual Jet Stream ESI interface. A reversed-phase analytical C18 column (Agilent Zorbax Eclipse Plus, 1.8 μm, 4.6 × 150 mm^2^) with a protective cartridge with the same packing was used. MS data were acquired in negative ionization mode, operating in full scan across a mass-to-charge ratio (*m*/*z*) range of 50–1700. The analytical method used was adapted from that described by Villegas-Aguilar et al.^19^

Analytical blank samples were injected at both the beginning and end of the analytical sequence. QC samples were analyzed right after the first blank samples for equilibration reasons. Furthermore, QCs were injected at regular intervals throughout the whole sequence, every six biological samples, to ensure analytical reproducibility. Biological samples from different experimental groups were randomized within the analytical sequences, considering that all plasma samples from the same participant were injected consecutively.

### 2.5 Data Preprocessing and Statistical Analysis

Initially, the acquired raw data were converted to a.mzML format using MSConvert. MZmine software (v 3.9.0) was employed to carry out several stages, including mass detection, ADAP Chromatogram Builder, ADAP Chromatogram deconvolution, alignment, isotope grouping, and gap filling. The following parameters were used for the ADAP chromatogram builder step: intensity threshold 3.0 × 10^2^; highest minimum intensity 1.0 × 10^3^; *m*/*z* tolerance 20 ppm. For chromatogram deconvolution: S/N threshold 10; minimum peak height 6.0E2; coefficient/area threshold 110; peak duration range 0.00–10.00; RT wavelet width range 0.00–0.10. The chromatograms were aligned using the “Join Aligner” algorithm with *m*/*z* and RT tolerances of 15 ppm and 0.25 min, respectively. After the processing steps, the resulting data set contained 22 598 molecular features, from which the information on the RT, *m*/*z*, and the corresponding areas of the different samples analyzed was obtained. Before applying the selection criteria for significant molecular features, those appearing in the analytical blanks were filtered out from the data set.

The following data processing steps were conducted using an open-source approach, incorporating several R packages. The batchCorr (v 0.2.5) R package was employed to normalize fluctuations due to within-batch and between-batches effects.^20^ The notame R package was utilized to remove biologically irrelevant signals, such as potential contaminants or those with low detection levels, using the *flag_contaminants* and *flag_detection* functions, respectively. Subsequently, the applied filters resulted in a data set comprising 6360 features. A principal component analysis (PCA) was performed using the MetaboAnalyst platform (version 6.0) to check data quality and identify possible outliers. Prior to PCA, the data were log-transformed and scaled by using Pareto scaling.

Subsequently, to identify statistically significant differences in signals between the experimental groups (placebo vs olive group; placebo vs lippia group), linear models with covariate adjustments were applied using the appropriate module in MetaboAnalyst software. The primary metadata included the experimental group with the placebo group set as the reference. The variable time was fixed as a covariate to account for variations in metabolite levels over time. A *p*-value cutoff of 0.05 was used, with the False Discovery Rate (FDR) correction applied to identify metabolites with significant differences between the groups. Considering mainly the placebo group and the samples taken for each volunteer at time 0, additional filtering criteria were applied to statistically significant variables to be certain that these signals correspond to exogenous metabolites whose origin is associated with the ingestion of the extract. The criteria associated with these additional filters are detailed below.

(1) The molecular features, in which the areas in the samples at baseline (time 0) were higher than the noise level (8 counts), were excluded.
(2) Molecular features were selected when the areas of at least one sample from each volunteer’s set of 7 plasma samples (0.5, 1, 2, 4, 6, 8, and 10 h), excluding the sample at baseline, were 10 times above the noise level (80 counts).
(3) The second condition must be met in a percentage higher than 50% of the volunteers who consumed either the OE or LC extracts. In contrast, this second condition should not be met in any volunteer in the placebo group.

Based on these criteria, a list of molecular features related to the potential circulating metabolites related to the studied extracts was obtained. These selected molecular features were proposed for annotation in the next stage. Targeted MS/MS analyses were conducted at different collision energies (10, 20, and 40 eV) on the significant features to generate fragmentation spectra for metabolite annotation. Then, the annotation was carried out by comparing the accurate mass, isotopic distribution, and MS/MS fragmentation spectra with information available in public databases (METLIN, FoodDB, HMDB, KEGG, and Pubchem) and mass banks. CEU Mass Mediator tool was used to search for potential candidates in the different mentioned databases.^21^ MS/MS spectra were also imported into other metabolomics annotation tools, such as Sirius^22^ or MetFrag (https://ipb-halle.github.io/MetFrag/), to search for potential annotated metabolites. Metabolites were annotated following the identification guidelines proposed by Sumner et al.^23^

Metabolite nutrikinetics were studied using PKSolver, an add-in program for pharmacokinetic data analysis in Microsoft Excel. The time to reach the maximum concentration (observed *T*_max_) was calculated for each significant metabolite. As an untargeted metabolomic approach was used to detect the circulating metabolites, these were not quantified. Therefore, the maximum concentration (*C*_max_) and area under the curve (AUC) parameters were calculated in a relative way by using the deconvoluted chromatographic areas. Statistical analyses to compare these nutrikinetic parameters between the two matrices were performed with GraphPad Prism version 8.01 (GraphPad Software, San Diego, CA). Statistical differences were determined by unpaired *t* tests. All *p* values less than 0.05 were considered statistically significant.

### 2.6 Quantification of Potential Precursor Phenolic Compounds of Common Bioavailable Metabolites in OE and LC Extracts

For a better interpretation of the potential common bioavailable metabolites, the phenolic compounds, verbascoside, oleuropein, hydroxytyrosol, and their derivatives present in the original extracts were quantified using an analytical method based on an HPLC-ESI-QTOF-MS platform.^18^ These compounds were quantified using calibration curves prepared with the corresponding analytical standards. Different dilutions of the analytical standards were prepared from a pool mix with a concentration of 100 mM per standard. Log-transformed data were used for the calibration models to adjust the exponential behavior detected between the concentration and peak areas. For each analytical standard, the calibration range, limit of detection (LOD), limit of quantification (LOQ), and the coefficient of determination (*R*^2^) were calculated (Table S1), showing good linearity (R^2^ > 0.99). LC and OE extracts at different dilutions (5000, 1000, 100, and 10 mg/L) were analyzed by the HPLC-ESI-QTOF-MS method to be able to quantify the phenolic compound present in high and low concentrations in the extracts. In the case of hydroxytyrosol- and oleuropein-derived compounds, the quantification of these compounds was tentatively performed using the calibration models of the hydroxytyrosol and oleuropein standards, respectively.

## 3 Results and Discussion

### 3.1 Phytochemical Composition of *L. citriodora* and *O. europaea* Extracts

The bioactive extracts of LC and OE used in the acute intervention study were tentatively characterized by HPLC-ESI-QTOF-MS in a previous study.^18^ As a summary of the phytochemical composition, 85 and 98 compounds were detected in the LC and OE extracts, respectively. In general, the LC extract was characterized by a particularly high content of phenylpropanoids. Within the phenylpropanoid group, verbascoside had the highest content, followed by its isoverbascoside isomer. In addition, a high content of iridoids and secoiridoids, such as shanziside and loganic acid, and glycosylated compounds of this type were also detected, such as gardoside. In the OE extract, the content of the glycosylated secoiridoid oleuropein was particularly high, as well as some derivative forms of these compounds, such as oleuropein-glucoside, methoxyoleuropein, hydroxyoleuropein, and oleuropein aglycone. Other compounds belonging to the flavonoid family, such as quercetin 3-O-rutinoside, were also detected in the OE extract.^14^

Among the characterized compounds in both extracts, only 10 compounds were annotated in both matrices (Table 1). Some of the compounds present in the two matrices were verbascoside, isoverbascoside, gluconic acid, malic acid, and fatty acids such as linolenic acid and linoleic acid. In general, it stands out that these common compounds are more abundant in the extract of LC than in that of OE. The low number of common compounds in the original composition of the plant extracts from both matrices supports their use to address the study hypothesis focused on common circulating metabolites originating from different chemical compounds present in the extracts.

**Table 1:** Common Compounds Characterized in *L. citriodora* and *O. europaea* Extracts

peak	RT^a^ (min)	observed [M – H]^−^	mol. formula	compound	relative area L. citriodora (relative units)	relative area O. europaea (relative units)
1	1.01	195.0511	C_6_H_12_O_7_	gluconic acid	1.3 × 10^7^	3.1 × 10^6^
2	1.07	133.0140	C_4_H_6_O_5_	malic acid	2.4 × 10^6^	9.8 × 10^6^
3	8.19	593.1497	C_27_H_30_O_15_	kaempferol 3-O-rutinoside	7.0 × 10^6^	4.6 × 10^5^
4	9.33	623.1978	C_29_H_36_O_15_	verbascoside	2.5 × 10^8^	4.0 × 10^6^
5	13.53	307.1920	C_18_H_28_O_4_	dihydrocapsiate	6.0 × 10^6^	2.0 × 10^5^
6	18.46	277.2159	C_18_H_30_O_2_	linolenic acid	1.5 × 10^7^	7.0 × 10^6^
7	18.73	375.2712	C_27_H_36_O	10′-apo-β-carotenal	2.4 × 10^4^	2.2 × 10^5^
8	19.16	279.2328	C_18_H_32_O_2_	linoleic acid	6.1 × 10^6^	3.7 × 10^6^
9	19.82	255.2325	C_16_H_32_O_2_	palmitic acid	9.9 × 10^6^	3.8 × 10^6^
10	19.95	281.2482	C_18_H_34_O_2_	oleic acid	2.7 × 10^6^	4.7 × 10^6^

a RT: retention time.

### 3.2 Selection of Significant Signals Related to the Intake of Supplements Using an Untargeted Metabolomic Methodology

Blood plasma samples were analyzed using an innovative untargeted metabolomics methodology based on HPLC-ESI-QTOF-MS to detect circulating compounds related to the intake of OE and LC extracts. After preprocessing the data with the specified filters, a total of 6360 molecular features were obtained. To assess data quality, an initial overview of the performance quality was obtained through PCA of the entire data set, including all QC samples (Figure S1). The PCA reveals well-clustered QC samples, indicating good data quality.

Following the application of linear models with covariate adjustments with an FDR of 0.05, a total of 740 signals were found to be statistically significant when comparing the olive group to the placebo group, while 756 signals were significant in the comparison between the Lippia group and placebo group. The high number of statistically significant signals is notable; however, it was observed that not all of these signals were associated with exogenous compounds. This is because the statistical analysis is sensitive to any changes in intensities between the groups, including potential changes in endogenous signals across different groups. Therefore, additional filtering stages were applied to ensure that the proposed signals for identification were associated with exogenous metabolites. These metabolites were related to the intake of bioactive supplements. Specifically, three additional criteria were applied, based primarily on the samples at t0 and in the placebo groups, as described in Section 2. After these three filtering criteria were applied, 71 signals associated with exogenous metabolites from the OE extracts and 43 signals from the LC extracts were selected for identification. Among the selected molecular features, nine were common to both matrices. The decrease in the number of signals selected in this last stage stands out, with all of those features filtered in the third criterion potentially related to other factors unrelated to the intake of bioactive supplements. Nevertheless, some molecular features related to the extracts could have been detected in less than 50% of the volunteers, possibly related to very low-concentration compounds, but they were not selected, as they were not reproducible in a significant proportion of the volunteers. Taking this criterion into account, the metabolites appearing in 8 volunteers (100%) among those selected will exhibit greater reproducibility and consequently biological importance, compared to those that appear in a smaller number of volunteers. Then, the selected 106 molecular features were proposed for annotation, of which 66 could be annotated. Among these 66 compounds, 26 and 48 were detected in the volunteers who intake the LC and OE extract (Tables 2 and 3), respectively. Nine of these 66 were common compounds detected in both LC and OE groups (Table 4). Out of the 66 compounds that were annotated, 3 were classified at level 1 after being verified against commercial analytical standards. Additionally, 46 compounds received a level 2 annotation based on a comparison of their MS/MS spectra with those listed in public databases or mass spectrometry libraries, while 17 compounds were assigned level 3 annotations, which relied on molecular mass and literature references. As a result, the majority of annotated compounds were at least categorized at level 2, reflecting a high degree of reliability in the annotations. The remaining 16 compounds were designated as level 3, indicating a lower level of reliability, yet the available bibliographic evidence points to a strong possibility of the identified compounds being relevant to the studied plant matrices. Furthermore, 39 molecular features could not be identified and were classified as unknown compounds (Table S2).

**Table 2:** HPLC-ESI-QTOF-MS Annotation Data and Relative Nutrikinetic Parameters of Metabolites Found in Plasma Samples Following Ingestion of a *L. citriodora* Extract^a^

RT (min)	[M – H]^−^	molecular formula	FDR	proposed compound	annotation level	MS/MS fragments	reference^b^	*n*	*C*_max_	observed *T*_max_	AUC
6.53	233.0122	C_8_H_10_O_6_S	6.41 × 10^–06^	hydroxytyrosol sulfate isomer 2	2	153.0597, 123.0441, 96.9549	(34)	7	902 ± 428	8 ± 2	3802 ± 2564
7.25	246.9916	C_8_H_8_O_7_S	2.31 × 10^–06^	vanillic acid 4-O-sulfate isomer 1	1^c^	123.0425, 167.0359, 78.9581, 96.9533	HMDB0041788	7	874 ± 644	8 ± 3	3576 ± 3122
8.92	329.0875	C_14_H_18_O_9_	4.90 × 10^–02^	hydroxytyrosol glucuronide isomer 2	1^d^	153.0543, 123.0435, 95.0112	(34)	4	106 ± 17	8 ± 2	350 ± 158
9.24	247.0279	C_9_H_12_O_6_S	4.80 × 10^–02^	homovanillyl alcohol sulfate	2	167.0706, 137.3427	(34)	4	156 ± 49	7 ± 2	437 ± 278
9.26	123.0450	C_7_H_8_O_2_	3.90 × 10^–02^	3-methylcatechol	2	121.0305	(34)	6	169 ± 59	8 ± 3	565 ± 407
9.69	373.1128	C_16_H_22_O_10_	3.07 × 10^–08^	gardoside isomer 1	2	211.0607, 122.8951	(35)	8	211 ± 72	2 ± 0	608 ± 375
9.81	261.0075	C_9_H_10_O_7_S	2.06 × 10^–05^	homovanillic acid sulfate isomer 1	1^e^	181.0494, 79.9585, 137.0609, 217.1051	HMDB0011719	7	341 ± 164	9 ± 2	1391 ± 1144
10.29	261.0071	C_9_H_10_O_7_S	1.89 × 10^–02^	homovanillic acid sulfate isomer 2	2	181.0495, 79.9587, 137.0619, 217.1055	HMDB0011719	5	476 ± 363	9 ± 2	1771 ± 1599
10.54	357.082	C_15_H_18_O_10_	6.99 × 10^–04^	homovanillic acid glucuronide	2	181.0501, 313.0923	(34)	6	360 ± 219	8 ± 2	1345 ± 1042
10.85	258.9912	C_9_H_8_O_7_S	1.89 × 10^–09^	caffeic acid 4-sulfate isomer 1	2	179.0352, 96.9537, 135.0447	HMDB0041708	7	325 ± 147	3 ± 1	1157 ± 757
11.28	258.9924	C_9_H_8_O_7_S	1.67 × 10^–07^	caffeic acid 4-sulfate isomer 2	2	179.0350, 96.953, 135.0452	HMDB0041708	6	357 ± 268	3 ± 2	1656 ± 1922
11.58	273.0049	C_10_H_10_O_7_S	1.27 × 10^–08^	ferulic acid 4-sulfate	2	193.0506, 96.9602	HMDB0240716	7	354 ± 254	3 ± 2	1752 ± 1781
12.37	369.0839	C_16_H_18_O_10_	4.70 × 10^–04^	ferulic acid 4-O-glucuronide	2	193.0510, 235.9254, 175.0228	HMDB0041733	5	282 ± 358	5 ± 2	1304 ± 1975
12.43	201.1130	C_10_H_18_O_4_	7.34 × 10^–03^	sebacic acid isomer	3		HMDB000079	5	119 ± 27	1.2 ± 0.4	141 ± 86
12.55	246.9907	C_8_H_8_O_7_S	7.81 × 10^–03^	vanillic acid 4-O-sulfate isomer 2	2	123.0423,167.0356, 96.9529	HMDB0041788	5	119 ± 41	1.2 ± 0.4	152 ± 118
13.75	373.1132	C_16_H_22_O_10_	4.55 × 10^–29^	gardoside isomer 2	2	211.0615, 122.8953	(35)	6	508 ± 110	2.3 ± 0.7	2015 ± 547
14.16	411.2032	C_21_H_32_O_8_	2.14 × 10^–04^	abscisic alcohol 11-glucoside isomer 1	2	75.0075, 55.0206, 307.1387	HMDB0039636	6	202 ± 82	1.2 ± 0.4	259 ± 134
16.12	343.1388	C_16_H_24_O_8_	7.07 × 10^–04^	dihydroconiferin isomer 1	2	181.0870	Pubchem: 14427336	6	139 ± 19	0.9 ± 0.2	126 ± 28
16.32	291.0863	C_15_H_16_O_6_	1.94 × 10^–04^	picrotoxinin	3	-	Pubchem: 442292	6	136 ± 44	1.3 ± 0.5	231 ± 102
16.43	343.1390	C_16_H_24_O_8_	1.84 × 10^–04^	dihydroconiferin isomer 2	2	181.0873	Pubchem: 14427336	5	136 ± 28	1 ± 0	244 ± 102
16.75	411.2010	C_21_H_32_O_8_	3.32 × 10^–09^	abscisic alcohol 11-glucoside isomer 2	2	75.0073, 55.0205, 307.1389	HMDB0039636	8	562 ± 251	2 ± 1	757 ± 174
17.44	411.2008	C_21_H_32_O_8_	4.52 × 10^–05^	abscisic alcohol 11-glucoside isomer 3	2	75.0069, 307.1393	HMDB0039636	7	258 ± 95	1 ± 0	267 ± 94
17.92	393.1899	C_21_H_30_O_7_	5.48 × 10^–03^	pteroside Z isomer 1	2	231.1387, 177.0312	HMDB32587	6	120 ± 22	1.2 ± 0.4	123 ± 53
18.05	393.1909	C_21_H_30_O_7_	4.43 × 10^–07^	pteroside Z isomer 2	2	231.1385	HMDB32587	8	181 ± 58	2 ± 1	310 ± 84
18.38	395.2051	C_21_H_32_O_7_	1.47 × 10^–08^	isopetasoside	3		HMDB29622	8	455 ± 162	2 ± 1	722 ± 166
19.17	293.2116	C_18_H_30_O_3_	1.41 × 10^–11^	17-hydroxylinolenic acid	3		HMDB00111	8	335 ± 306	6 ± 4	1621 ± 1914
average									307 ± 210	4 ± 3	997 ± 970

a *n*: number of volunteers in which the metabolite was detected after intake of the *L. citriodora* extract; RT: retention time; *C*_max_: relative maximum plasma level (relative chromatographic area); observed *T*_max_: time required to reach *C*_max_ (h); AUC: area under the zero-moment curve (relative chromatographic area/h); values represent mean ± standard deviation (SD).

b Metabolomic databases or bibliographic references utilized for annotation.

c MS/MS spectra of vanillic acid 4-O-sulfate annotated at level 1 in Figure S2.

d MS/MS spectra of hydroxytyrosol glucuronide annotated at level 1 in Figure S3.

e MS/MS spectra of homovanillic acid sulfate annotated at level 1 in Figure S4.

**Table 3:** HPLC-ESI-QTOF-MS Annotation Data and Relative Nutrikinetic Parameters of Metabolites Found in Plasma Samples Following the Ingestion of a*O. europaea* Extract^a^

RT (min)	[M – H]^−^	molecular formula	FDR	proposed compound	annotation level	MS/MS fragments	reference^b^	*n*	*C*_max_	observed *T*_max_	AUCt
6.41	233.0124	C_8_H_10_O_6_S	9.53 × 10^–06^	hydroxytyrosol sulfate isomer 1	2	153.0599, 123.0443, 96.9557	(34)	6	593 ± 382	3 ± 3	1755 ± 1392
6.53	233.0122	C_8_H_10_O_6_S	2.60 × 10^–12^	hydroxytyrosol sulfate isomer 2	2	153.0597, 123.0441, 96.9549	(34)	8	836 ± 359	3 ± 3	2914 ± 1792
7.25	246.9916	C_8_H_8_O_7_S	2.81 × 10^–18^	vanillic acid 4-O-sulfate isomer 1	1^c^	123.0425,167.0359, 78.9581, 96.9533	HMDB0041788	8	489 ± 147	1.0 ± 0.0	2361 ± 729
8.65	329.0875	C_14_H_18_O_9_	1.19 × 10^–23^	hydroxytyrosol glucuronide isomer 1	2	153.0541, 123.0433, 95.0115	(34)	8	1002 ± 411	0.9 ± 0.2	2266 ± 993
8.92	329.0875	C_14_H_18_O_9_	1.74 × 10^–34^	hydroxytyrosol glucuronide isomer 2	1^d^	153.0543, 123.0435, 95.0112	(34)	8	2627 ± 1167	0.9 ± 0.2	6846 ± 2440
8.93	351.0697	C_16_H_16_O_9_	3.48 × 10^–12^	chlorogenoquinone	2	175.0318	HMDB0029383	8	324 ± 7	0.9 ± 0.2	605 ± 254
9.24	247.0279	C_9_H_12_O_6_S	4.59 × 10^–05^	homovanillyl alcohol sulfate	2	167.0706, 137.3427	(34)	8	135 ± 32	2.0 ± 0.9	360 ± 155
9.26	123.0450	C_7_H_8_O_2_	1.09 × 10^–05^	3-methylcatechol	2	121.0305	(34)	7	307 ± 160	0.6 ± 0.2	585 ± 562
9.75	343.0686	C_14_H_16_O_10_	2.76 × 10^–04^	vanillic acid glucuronide	2	255.0046	HMDB0060024	7	111 ± 48	4 ± 3	351 ± 232
9.81	261.0075	C_9_H_10_O_7_S	5.94 × 10^–16^	homovanillic acid sulfate isomer 1	1^e^	181.0494, 79.9585, 137.0609, 217.1051	HMDB0011719	8	233 ± 69	3 ± 2	1338 ± 674
10.10	343.1033	C_15_H_20_O_9_	4.27 × 10^–23^	homovanillyl alcohol glucuronide isomer 1	2	167.0614, 135.0423	HMDB0240527	8	504 ± 154	1.1 ± 0.3	1623 ± 620
10.28	259.0819	C_11_H_16_O_7_	1.98 × 10^–14^	3-furanmethanol glucoside isomer 1	2	165.0562, 121.0648, 241.0689, 95.0823	HMDB0032924	8	236 ± 104	1.6 ± 0.5	688 ± 337
10.29	261.0071	C_9_H_10_O_7_S	3.73 × 10^–02^	homovanillic acid sulfate isomer 2	2	181.0495, 79.9587, 137.0619, 217.1055	HMDB0011719	8	215 ± 68	4 ± 2	1484 ± 597
10.52	181.0867	C_10_H_14_O_3_	2.57 × 10^–10^	1,2,3-trimethoxy-5-methyl benzene	3		(40)	8	221 ± 72	1 ± 0	384 ± 150
10.53	149.0604	C_9_H_10_O_2_	4.26 × 10^–08^	*p*-vinylguaiacol	3		(41)	8	184 ± 45	1 ± 0	320 ± 138
10.54	225.0753	C_11_H_14_O_5_	1.23 × 10^–04^	desoxy elenolic acid	3		(42)	6	124 ± 24	1.1 ± 0.4	174 ± 85
10.54	357.0820	C_15_H_18_O_10_	6.65 × 10^–08^	homovanillic acid glucuronide	2	181.0501; 313.0923	(34)	7	199 ± 54	4 ± 1	1091 ± 562
10.55	243.0870	C_11_H_16_O_6_	2.33 × 10^–16^	threo-syringoylglycerol isomer 1	2	123.0818, 211.0603,167.0707	HMDB0031237	8	442 ± 138	1 ± 0	1001 ± 389
10.60	211.0606	C_10_H_12_O_5_	8.44 × 10^–05^	eudesmic acid	2	167.0708	FDB012013	6	120 ± 27	1 ± 0	150 ± 93
11.06	411.0904	C_22_H_20_O_6_S	1.25 × 10^–07^	4β-benzylthioepicatechin	2	242.8023	(43)	7	158 ± 62	1.7 ± 0.5	432 ± 270
11.09	343.1032	C_15_H_20_O_9_	1.64 × 10^–31^	homovanillyl alcohol glucuronide isomer 2	2	167.0616, 135.0425	HMDB0240527	8	735 ± 257	1.6 ± 0.5	2867 ± 1078
11.24	259.0819	C_11_H_16_O_7_	5.54 × 10^–38^	3-furanmethanol glucoside isomer 2	2	165.0563, 121.0649, 241.0692, 95.0827	HMDB0032924	8	1234 ± 489	1 ± 0	4372 ± 1633
11.24	327.0695	C_14_H_16_O_9_	5.70 × 10^–11^	vanillin glucuronide	3		HMDB0240573	8	180 ± 66	1 ± 0	448 ± 297
11.58	273.0049	C_10_H_10_O_7_S	4.25 × 10^–02^	ferulic acid 4-sulfate	2	193.0506, 96.9602	HMDB0240716	5	142 ± 20	1.6 ± 0.5	267 ± 218
12.13	229.0714	C_12_H_22_O_4_	1.48 × 10^–16^	decanedioic acid isomer 1	3		0975-5071	8	232 ± 48	2 ± 0	967 ± 366
12.40	229.0712	C_12_H_22_O_4_	1.03 × 10^–04^	decanedioic acid isomer 2	3		0975-5071	5	112 ± 11	6 ± 3	438 ± 201
12.75	275.0229	C_10_H_12_O_7_S	3.62 × 10^–52^	dihydroferulic acid 4-sulfate	3		HMDB0041724	8	190 ± 20	1.3 ± 0.4	297 ± 238
13.09	371.0979	C_16_H_20_O_10_	3.43 × 10^–18^	dihydroferulic acid 4-O-glucuronide	3		HMDB0041723	8	171 ± 110	2 ± 2	896 ± 861
13.64	243.0869	C_11_H_16_O_6_	1.22 × 10^–21^	threo-syringoylglycerol isomer 2	2	123.0821, 211.0603,167.0705	HMDB0031237	8	568 ± 141	0.8 ± 0.2	1393 ± 460
13.90	243.0868	C_11_H_16_O_6_	1.09 × 10^–16^	threo-syringoylglycerol isomer 3	2	123.0819, 211.0601,167.0709	HMDB0031237	8	414 ± 105	0.8 ± 0.2	939 ± 302
13.91	555.1712	C_25_H_32_O_14_	5.31 × 10^–07^	hydroxyoleuropein isomer 1	2	393.0907,323.0458	(38)	8	524 ± 281	0.9 ± 0.5	690 ± 401
13.95	553.1556	C_25_H_30_O_14_	1.36 × 10^–07^	oleuropein aglycone glucuronide isomer 1	2	377.1260, 275.1608, 165.0559	(34)	8	244 ± 128	0.8 ± 0.2	289 ± 205
13.97	457.0805	C_22_H_18_O_11_	5.27 × 10^–04^	epigallocatechin 7-O-gallate	2	305.0661	HMDB0003153	7	126 ± 30	1 ± 0	120 ± 53
13.97	593.1486	C_27_H_30_O_15_	1.41 × 10^–03^	vicenin-2	3		HMDB0030708	5	137 ± 59	1 ± 0	190 ± 135
14.05	555.1709	C_25_H_32_O_14_	1.66 × 10^–17^	hydroxyoleuropein isomer 2	2	393.0903, 323.0456	(38)	8	865 ± 264	0.9 ± 0.2	1754 ± 511
14.05	623.1584	C_28_H_32_O_16_	7.96 × 10^–04^	isorhamnetin 3-O-glucoside-7-O-rhamnoside	3		Pubchem: 72188972	6	133 ± 18	0.9 ± 0.2	137 ± 65
14.07	585.1815	C_26_H_34_O_15_	1.10 × 10^–03^	10-hydroxy-7 -methoxyoleuropein isomer 1	2	409.1498, 113.0227, 176.0274	(44)	5	107 ± 22	2 ± 0	240 ± 113
14.11	541.1562	C_27_H_42_O_11_	1.55 × 10^–03^	cortolone-3-glucuronide	2	175.0223	HMDB0010320	5	163 ± 88	4.4 ± 0.8	610 ± 515
14.16	553.1558	C_25_H_30_O_14_	4.25 × 10^–13^	oleuropein aglycone glucuronide isomer 2	2	377.1262, 275.1612, 165.0560	(34)	8	549 ± 230	0.9 ± 0.2	905 ± 398
14.26	585.1817	C_26_H_34_O_15_	1.56 × 10^–14^	10-hydroxy-7 -methoxyoleuropein isomer 2	2	409.1499, 113.0230, 176.0275	(44)	8	241 ± 89	1.9 ± 0.3	837 ± 406
14.27	569.1868	C_26_H_34_O_14_	2.81 × 10^–03^	methoxyoleuropein isomer 1	2	529.1235; 551.657	HMDB0035445	6	147 ± 31	1.5 ± 0.5	209 ± 82
14.95	555.1712	C_25_H_32_O_14_	7.32 × 10^–09^	hydroxyoleuropein isomer 3	2	393.0906, 323.0457	(38)	8	471 ± 143	0.7 ± 0.2	601 ± 318
15.07	569.1870	C_26_H_34_O_14_	1.23 × 10^–07^	methoxyoleuropein isomer 2	2	529.1237, 551.653	HMDB0035445	6	181 ± 49	0.7 ± 0.2	553 ± 433
15.28	555.1714	C_25_H_32_O_14_	1.48 × 10^–03^	hydroxyoleuropein isomer 4	2	393.0917, 329.0459	(38)	5	359 ± 121	0.8 ± 0.2	339 ± 196
15.49	569.1867	C_26_H_34_O_14_	1.09 × 10^–16^	methoxyoleuropein isomer 3	2	529.1229, 551.661	HMDB0035445	8	794 ± 449	0.9 ± 0.2	1745 ± 1341
15.56	364.9974	C_15_H_10_O_9_S	1.10 × 10^–04^	kaempferol sulfate	2	151.0037, 96.9557	(45)	7	172 ± 102	1.1 ± 0.3	226 ± 186
15.77	201.1130	C_10_H_18_O_4_	8.10 × 10^–07^	debacic acid isomer 3	3		HMDB000079	7	330 ± 109	6 ± 2	1628 ± 518
15.90	393.1184	C_20_H_26_O_8_	3.83 × 10^–07^	10-hydroxyoleuropein aglycone	3		(46)	8	194 ± 76	0.7 ± 0.2	214 ± 126
22.37	535.3086	C_33_H_44_O_6_	5.90 × 10^–05^	dihydrocelastryl diacetate	3		(47)	5	239 ± 78	3 ± 1	837 ± 571
average									392 ± 417	2 ± 1	1072 ± 1208

a *n*: number of volunteers in which the metabolite was detected after intake of the *O. europaea* extract; RT: retention time; *C*_max_: relative maximum plasma level (relative chromatographic area); observed *T*_max_: time required to reach *C*_max_ (h); AUC: area under the zero-moment curve (relative chromatographic area/h); values represent mean ± SD.

b Metabolomic databases or bibliographic references utilized for annotation.

c MS/MS spectra of vanillic acid 4-O-sulfate annotated at level 1 in Figure S2.

d MS/MS spectra of hydroxytyrosol glucuronide annotated at level 1 in Figure S3.

e MS/MS spectra of homovanillic acid sulfate annotated at level 1 in Figure S4.

**Table 4:** Nutrikinetic Parameters and Statistical Analysis of Common Metabolites after Ingestion of an Extract of *L. citriodora* and *O. europaea*^a^

	*n*		*C*_max_			observed *T*_max_			AUC		
proposed compound	LC	OE	LC	OE	*p*-value	LC	OE	*p*-value	LC	OE	*p*-value
hydroxytyrosol sulfate isomer 2	7	8	902 ± 428	836 ± 359	7.5 × 10^–01^	8 ± 2	3 ± 3	7.0 × 10^–04^^b^	3802 ± 2564	2914 ± 1792	4.5 × 10^–01^
vanillic acid 4-O-sulfate isomer 1	7	8	874 ± 644	489 ± 147	1.2 × 10^–01^	8 ± 3	1.0 ± 0.0	8.0 × 10^–07^^b^	3576 ± 3122	2361 ± 729	3.0 × 10^–01^
hydroxytyrosol glucuronide isomer 2	4	8	106 ± 17	2627 ± 1167	1.8 × 10^–03^^b^	8 ± 2	0.9 ± 0.2	1.0 × 10^–06^^b^	350 ± 158	6846 ± 2440	4.0 × 10^–04^^b^
homovanillyl alcohol sulfate	4	8	156 ± 49	135 ± 32	3.9 × 10^–01^	7 ± 2	2.0 ± 0.9	2.0 × 10^–04^^b^	437 ± 278	360 ± 155	5.4 × 10^–01^
3-methylcatechol	6	7	169 ± 59	307 ± 160	7.2 × 10^–02^	8 ± 3	0.6 ± 0.2	5.0 × 10^–05^^b^	565 ± 407	585 ± 562	9.4 × 10^–01^
homovanillic acid sulfate isomer 1	7	8	341 ± 164	233 ± 69	1.1 × 10^–01^	9 ± 2	3 ± 2	1.0 × 10^–04^^b^	1391 ± 1144	1338 ± 674	9.1 × 10^–01^
homovanillic acid sulfate isomer 2	5	8	476 ± 363	215 ± 68	6.7 × 10^–02^	9 ± 2	4 ± 2	4.0 × 10^–05^^b^	1771 ± 1599	1484 ± 597	6.5 × 10^–01^
homovanillic acid glucuronide	6	7	360 ± 219	199 ± 54	1.1 × 10^–01^	8 ± 2	4 ± 1	5.0 × 10^–04^^b^	1345 ± 1042	1091 ± 562	6.1 × 10^–01^
ferulic acid 4-sulfate	7	5	354 ± 254	142 ± 20	9.4 × 10^–02^	3 ± 2	1.6 ± 0.5	9.2 × 10^–02^	1752 ± 1781	267 ± 218	9.5 × 10^–02^

a *n*: number of volunteers in which the metabolite was detected following the ingestion of *Lippia citriodora* (LC) or *Olea europaea* (OE) extracts. *C*_max_: relative maximum plasma level (relative chromatographic area); observed *T*_max_: time required to reach *C*_max_ (h); AUC: area under the zero-moment curve (relative chromatographic area/h); values represent mean ± standard deviation (SD).

b Statistically significant differences (*p* < 0.05) between the nutrikinetic parameters of LC and OE.

### 3.3 Metabolites Detected in Plasma Associated with *L. citriodora* Intake

Table 2 presents the annotation and nutrikinetic parameters of metabolites detected in plasma samples following the ingestion of an LC extract. More information is reported in Table S3, where the means of the chromatographic areas of these compounds for different times are detailed.

**Figure 1:**
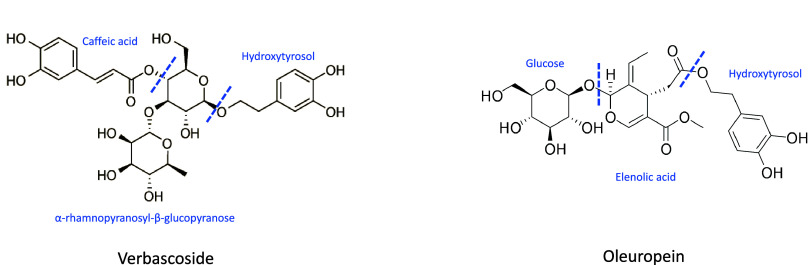
Chemical structures of verbascoside and oleuropein.

Among these compounds, two isomers of gardoside, a compound that appeared in the original extract, were tentatively identified with MS^2^ fragments *m*/*z* 211.0607 ([M – glucose]^−^) and 122.8951 ([M – glucose-88]^−^), suggesting that this compound could be absorbed in its native form. In a study in which LC extract was administered to rats, the presence of gardoside in its native form in plasma was also observed, which is consistent with our results in humans.^10^ It is noteworthy that verbascoside, despite being one of the major compounds characterized in the LC extract, has not been detected in plasma samples either in its native form or in its possible phase II metabolites. However, compounds resulting from the cleavage of verbascoside and modifications resulting from phase II metabolic reactions have been detected. These compounds are caffeic acid 4-sulfate (with MS^2^ fragments *m*/*z* 179.0352 (C_9_H_7_O_4_) and 135.0447 (C_8_H_7_O_2_)), ferulic acid 4-O-glucuronide (with MS^2^ fragments *m*/*z* 193.0510 (C_10_H_9_O_4_), 235.9254 (C_12_H_11_O_5_) and 175.0228 (C_10_H_7_O_3_)) and vanillic acid 4-O-sulfate (with MS^2^ fragments *m*/*z* 123.0425 (C_7_H_7_O_2_), 167.0359 (C_8_H_7_O_4_), 78.9581 (C_5_H_3_O) and 96.9533 (HO_4_S)). Although these three metabolites could potentially derive from their respective acids (caffeic, ferulic, and vanillic acids), they are not present in the original composition of the LC extract. This rules out this possible origin and indicates that their presence is likely related to the metabolization of verbascoside. Verbascoside is easily fragmented into caffeic acid,^24^ and from this by methylation modification or fragmentation can be derived to hydroxytyrosol, ferulic acid, and vanillic acid^25−27^ (Figure 1). Some of these compounds, such as caffeic acid, which has been identified in its sulfate form, and the glucuronide forms of ferulic acid have been identified in previous bioavailability studies in rats. This consistency aligns with the results obtained in the current study.^10^

Similar observations apply to other compounds, such as various isomers of abscisic alcohol 11-glucoside (with MS^2^ fragments *m*/*z* 75.0075 (C_2_H_3_O_3_), 55.0206 (C_4_H_7_), and 307.1387 (C_17_H_23_O_5_)), which appear in plasma but were not present in the original LC extract. These compounds found in plasma may originate from the fragmentation of other compounds. In the case of abscisic alcohol 11-glucoside, it may also represent a fragment derived from the metabolism of more complex phenolic compounds, a phenomenon observed in other studies involving the consumption of foods rich in phenolic compounds.^28^

When the *T*_max_ of the different metabolites present in plasma is analyzed, in the case of the volunteers who ingested LC (Table 2), compounds including gardoside isomers, caffeic acid 4-sulfate isomers, and ferulic acid 4-sulfate show a maximum observed *T*_max_ around 2–3 h after ingestion of the extract. However, other compounds such as hydroxytyrosol sulfate isomer 2, vanillic acid 4-o-sulfate isomer 1, hydroxytyrosol glucuronide isomer 2, homovanillyl alcohol sulfate, 3-methylcatechol, two isomers of homovanillic acid sulfate, and homovanillic acid glucuronide have the highest observed *T*_max_ values between 8 and 10 h after ingestion of the extract. This may be explained by the fact that the compounds that appear earlier (at 2 h) are compounds that appear after fewer metabolic reactions. In this sense verbascoside is split into caffeic acid and hydroxytyrosol in phase I metabolism^29^ and in phase II metabolism these compounds are sulfated. In the case of gardoside, it is found in the original extract and therefore directly undergoes the sulfation reaction. Caffeic acid is easily transformed into ferulic acid by the enzyme caffeate O-methyltransferase and then passed on.^30^ Most of the hydroxytyrosol is transformed into the sulfated form, as the AUC is about 10 times higher at the peak compared to the glucuronidated form, which is also excreted much later. These results are in agreement with those obtained in another study in which they evaluated oral bioavailability and metabolism of hydroxytyrosol from food supplements and found that hydroxytyrosol in its sulfated form was more than 10 times higher than the glucuronidated form.^31^

**Figure 2:**
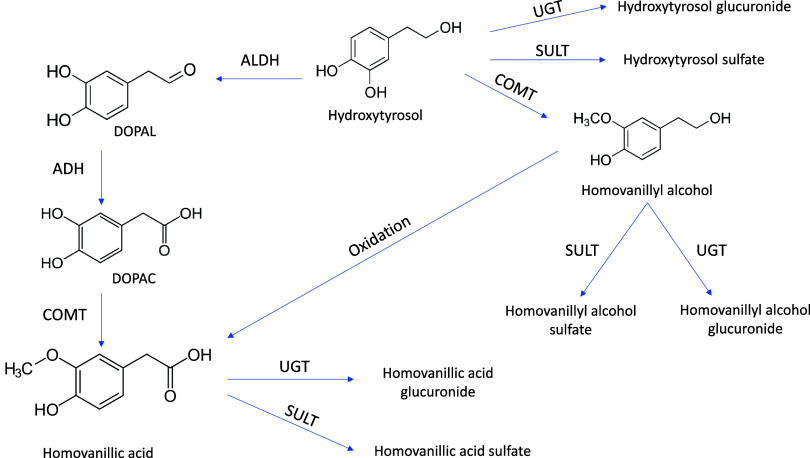
Hydroxytyrosol metabolic pathways. ADH: alcohol dehydrogenase; ALDH: aldehyde dehydrogenase; COMT: catechol-O-methyltransferase; DOPAC: 3,4-dihydroxyphenylacetic acid; DOPAL: 3,4-dihydroxyphenylacetaldehyde; SULT: sulfotransferase; UGT: UDP-glucuronosyl transferase.

The other compounds that show the observed *T*_max_ after 6–8 h after ingestion undergo more metabolic transformations, so it is reasonable to conclude that they take longer to appear. For example, homovanillyl alcohol sulfate (with MS^2^ fragments *m*/*z* 167.0706 (C_9_H_11_O_3_) and 137.3427 (C_8_H_9_O_2_)), can appear in plasma from hydroxytyrosol, which by catechol-O-methyltransferase gives rise to homovanillyl alcohol and sulfotransferase gives rise to homovanillyl alcohol sulfate (Figure 2). In the case of homovanillic acid sulfate and homovanillic acid glucuronide, hydroxytyrosol via aldehyde dehydrogenase gives rise to 3,4-dihydroxyphenylacetaldehyde (DOPAL) and this compound via alcohol dehydrogenase gives rise to 3,4-dihydroxyphenylacetic acid (DOPAC), which is finally transformed to homovanillic acid by catechol-O-methyltransferase. Finally, this homovanillic acid may undergo sulfation or glucuronidation in phase II metabolism. Moreover, vanillic acid can be formed from ferulic acid by transforming it to vanillin and then to vanillic acid, by oxidation reactions or it can be transformed by bacteria present in the microbiota such as *Paraburkholderia aromaticivora* via vanillin.^32,33^

### 3.4 Metabolites Detected in Plasma Associated with *O. europaea* Intake

Table 3 presents the annotation and nutrikinetic parameters of metabolites detected in plasma samples following the ingestion of an *Olea europaea* extract. In addition, Table S4 presents the mean values of the chromatographic areas for each compound detected in the *OE* group.

Although oleuropein is the major compound in the original extract, it was not detected as being significant in plasma samples. This can be explained by the fact that the compound was detected in plasma, but it was also detected in the volunteers in the placebo group, and therefore, it was not selected as significant. This fact could be explained by the fact that olive oil is widely consumed in the Mediterranean region, where the study was carried out.^36^ Therefore, oleuropein in the plasma of all volunteers was found at basal levels, well below the values found by the consumption of the extract. In the case of the compounds present only in plasma samples from volunteers who ingested OE, the following different isomers of four oleuropein-derived structures were detected: four isomers of hydroxyoleuropein, three isomers of oleuropein aglycone glucuronide, two isomers of 10-hydroxy-7-methoxyoleuropein, and three isomers of methoxyoleuropein. These oleuropein-derived compounds result from enzymatic hydrolysis and phase I and II metabolic reactions. Some of these compounds have been also detected in humans due to the consumption of olive oil, such as oleuropein aglycone glucuronide.^34^

Hydroxytyrosol, one of the most characteristic phenolic compounds in olive leaves, was also detected in plasma samples of the volunteers who consumed the OE extract. The presence of hydroxytyrosol in plasma confirms the bioavailability of this compound, which is intriguing because the extensive array of bioactive properties associated with this compound can be exerted *in vivo* within an organism.^37^ In addition, some metabolized compounds derived from hydroxytyrosol were also detected in plasma, such as hydroxytyrosol glucuronide (with MS^2^ fragments *m*/*z* 153.0541 (C_8_H_9_O_3_), 123.0433 (C_7_H_7_O_2_), and 95.0115 (C_6_H_7_O)) and hydroxytyrosol sulfate (with MS^2^ fragments *m*/*z* 153.0599 (C_8_H_9_O_3_), 123.0443 (C_7_H_7_O_2_), and 96.9557 (HO_4_S)). This compound may come either from the native form present in the extract or from the oleuropein-derived form, as it is part of its structure and by enzymatic hydrolysis can give rise to the free compound^38^ (Figure 1).

Two isomers of homovanillyl alcohol glucuronide (with MS^2^ fragments *m*/*z* 167.0614 (C_9_H_11_O_3_) and 135.0423 (C_8_H_7_O_2_)) were also detected in plasma. This compound is a metabolite derived from hydroxytyrosol (Figure 2). This form without the glucuronide^39^ has been detected in other studies in rats focused on the bioavailability of the metabolites of hydroxytyrosol. Therefore, it is worth noting that in our study in humans, the homovanillyl alcohol form does not appear but rather its glucuronide form in volunteers who ingested OE. Another metabolite derived from phase II metabolism that appeared in plasma samples was kaempferol sulfate (with MS^2^ fragments *m*/*z* 151.0037 (C_7_H_3_O_4_) and 96.9557 (HO_4_S)). This metabolite is the result of the metabolic sulfation reaction of kaempferol, which is a compound that was characterized in the original extract.

When examining the observed *T*_max_ of the metabolites that appeared in the volunteers who ingested OE, in contrast to the metabolites in LC, most of them show their observed *T*_max_ in the time period of 1–2 h after the ingestion of the extract. This may be because the main metabolites that appear either were in the original extract as different isomers of hydroxyoleuropein and methoxyoleuropein or compounds that were in the extract and undergo direct phase II metabolic reactions; for example, both oleuropein aglycone and hydroxytyrosol are in the original extract so the sulfated and glucuronidated forms of these that appear in plasma may result from these direct reactions.

When examining the mean values for all *C*_max_, observed *T*_max_, and AUC for the metabolites resulting from the ingestion of both extracts (Tables 2 and 3), it is generally observed that the mean values for these three variables are 307 ± 210; 4 ± 3; and 997 ± 970, respectively, for LC, while for OE, they are 392 ± 417; 2 ± 1; and 1072 ± 1208, respectively. After conducting a Student’s *t-*test, it was noted that only in the case of the observed *T*_max_, the mean values were significantly different. Therefore, similar to the metabolites shared by both matrices, there is an observed trend for metabolites from the LC extract to manifest approximately 2 h later on average compared to those from OE.

### 3.5 Circulating Metabolites Related to the Intake of Both Matrices

The nine common bioavailable metabolites (hydroxytyrosol sulfate, vanillic acid sulfate, hydroxytyrosol glucuronide, homovanillyl alcohol sulfate, 3-methylcatechol, homovanillic acid glucuronide, ferulic acid 4-sulfate, and two isomers of homovanillic acid sulfate) detected in plasma for both extracts are detailed in Table 4 along with their calculated nutrikinetic parameters.

Based on these results, the hypothesis of the origin of the most common bioavailable metabolites lies in the common origin related to the compound hydroxytyrosol (Figure 2). This compound is present only in the OE extract. However, the compounds verbascoside, present in LC and OE extracts, as well as oleuropein and its derivatives (e.g., hydroxyoleuropein, methoxyoleuropein isomer, oleuropein aglycone, etc.), present in OE, all contain a hydroxytyrosol unit in their structure (Figure 1). For this reason, all of these sources of hydroxytyrosol in both OE and LC extracts were quantified for better interpretation and discussion of the results achieved for the common metabolites. The quantification results of the potential precursor compounds of common circulating metabolites are shown in Table 5. These quantification results show that the OE extract, apart from having many more precursors than the LC extract, also has a higher total concentration of hydroxytyrosol equivalents. Although verbascoside is present in the composition of both extracts, the concentration in the OE extract is much lower than that in the LC extract. Therefore, it is demonstrated that the origin of the bioavailable common compounds is not solely due to the common phenolic compound in the original extracts.

**Table 5:** Quantification of Potential Precursor Compounds of Common Circulating Metabolites in *L. citriodora* and *O. europaea* Extracts^a^

compound	*m*/*z* [M – H]^−^	LC content (μmol/g dry extract)	LC content (%g/g dry extract)	OE content (μmol/g dry extract)	OE content (%g/g dry extract)
hydroxytyrosol isomer 1	153.0553	ND	ND	10 ± 1	0.15 ± 0.02
hydroxytyrosol isomer 2	153.0556	0.14 ± 0.03	0.0021 ± 0.0005	16.3 ± 0.7	0.25 ± 0.01
hydroxyoleuropein isomer 1	555.1715	ND	ND	0.52 ± 0.08	0.029 ± 0.004
hydroxytyrosol acetate	195.0660	ND	ND	10 ± 1	0.19 ± 0.02
demethyloleuropein	525.1608	ND	ND	0.091 ± 0.003	0.0047 ± 0.0002
hydroxyoleuropein isomer 2	555.1729	ND	ND	<LOQ	<LOQ
verbascoside isomer 1	623.1990	195 ± 36	12 ± 2	8.4 ± 0.3	0.52 ± 0.02
oleuropein-glucoside isomer 1	701.2302	ND	ND	4.1 ± 0.4	0.29 ± 0.03
verbascoside isomer 2	623.1988	2.84 ± 0.02	0.177 ± 0.001	0.039 ± 0.006	0.0024 ± 0.0004
oleuropein-glucoside isomer 2	701.2309	ND	ND	0.24 ± 0.06	0.017 ± 0.004
verbascoside isomer 3	623.1986	21.1 ± 0.9	1.32 ± 0.06	0.99 ± 0.05	0.062 ± 0.003
oleuropein-glucoside isomer 3	701.2294	ND	ND	0.169 ± 0.002	0.0118 ± 0.0001
methoxyoleuropein isomer 1	569.1883	ND	ND	1.04 ± 0.07	0.059 ± 0.004
oleuropein isomer 1	539.1783	ND	ND	258 ± 37	14 ± 2
methoxyoleuropein isomer 2	569.1895	ND	ND	0.0090 ± 0.0001	0.00051 ± 0.00005
oleuropein isomer 2	539.1782	ND	ND	8.4 ± 0.7	0.45 ± 0.04
4″-methyloleuropein	553.1935	ND	ND	0.014 ± 0.003	0.0008 ± 0.0001
oleuropein aglycone isomer 1	377.1244	ND	ND	0.018 ± 0.007	0.0007 ± 0.0003
10-hydroxyoleuropein aglycone isomer 1	393.1197	ND	ND	0.16 ± 0.01	0.0063 ± 0.0004
10-hydroxyoleuropein aglycone isomer 2	393.1189	ND	ND	0.594 ± 0.008	0.0234 ± 0.0003
oleuropein aglycone isomer 2	377.1244	ND	ND	9 ± 1	0.34 ± 0.04
total (μmol hydroxytyrosol equiv/g dry extract)		219 ± 37		328 ± 42	
% total			14 ± 2		16 ± 2

a LC: *L. citriodora*; OE: *O. europaea*; ND: not detectable; LOQ: limit of quantification.

Interestingly, despite the OE extract exhibiting a greater abundance of precursors for metabolites derived from hydroxytyrosol, verbascoside, and oleuropein (Table 5), the average AUC values between the two matrices were not statistically significant. This global trend based on the means of all compounds detected in both matrices is also consistent with the lack of significant differences in the *C*_max_ and AUC parameters for the 9 common metabolites, except for hydroxytyrosol glucuronide (Table 4).

In addition to the reported 9 common metabolites, three other common compounds were detected but were significant in fewer than 50% of volunteers who took the LC extract. Specifically, these metabolites were another isomer of hydroxytyrol sulfate, vanillic acid glucuronide, and sebacic acid, which were only detected in three, two, and three volunteers, respectively, who took the LC extract of the total of 8 volunteers. However, these compounds were considered in the analysis of the volunteers who consumed OE, as they were present in more than 50% of these volunteers. These differences can be attributed to the lower concentration of hydroxytyrosol precursors in the LC extract compared to that in the OE extract, as reported in Table 5.

The immediate metabolites resulting from phase II reactions involving hydroxytyrosol were identified as hydroxytyrosol sulfate and a hydroxytyrosol glucuronide isomer. It was observed that hydroxytyrosol sulfate may be derived from verbascoside, as in the case of LC, or from the free or oleuropein-derived hydroxytyrosol form, related to the OE extract. A relevant result is that a single isomer of the hydroxytyrosol glucuronide was detected to be common between the two matrices. However, another isomer of hydroxytyrosol glucuronide was detected exclusively in the plasma of volunteers who consumed OE. Furthermore, the common hydroxytyrosol glucuronide isomer exhibited substantially higher *C*_max_ and AUC in OE compared to those in LC extracts. These observations are likely attributable to the appreciably higher levels of precursors for this compound in OE extracts, as reported in Table 5. The presence of two isomers of hydroxytyrosol in the plasma samples of OE volunteers may also be because the free form of hydroxytyrosol in this matrix makes it more likely to undergo metabolism by different hydroxyl groups, resulting in both isomers being found in plasma.^48^ An alternative hypothesis to explain this difference could be that it is due to the steric hindrance posed by hydroxytyrosol in the verbascoside structure that prevents it from being metabolized by different OH groups. Nevertheless, there is limited information in the current literature to substantiate this hypothesis.

The presence of vanillic acid 4-O-sulfate, homovanillyl alcohol sulfate, homovanillic acid sulfate, homovanillic acid glucuronide, ferulic acid 4-sulfate and 3-methylcatechol in plasma samples of both groups may be due to the action of enzymatic hydrolysis and metabolic phase I and II reactions. In this context, the hydroxytyrosol derived from both compounds, through the action of catechol-O-methyltransferase, gives rise to homovanillic acid and alcohol derivatives. Subsequently, by the actions of UDP-glucuronosyltransferases and sulfotransferases, the compounds homovanillic acid glucuronide, homovanillyl alcohol sulfate, and homovanillic acid sulfate can be produced.^49^ 3-Methylcatechol is also a derivative of hydroxytyrosol which has also been detected in other bioavailability studies focused on olive oil, which is a product derived from the fruit of OE.^34^

It should be noted that ferulic acid 4-sulfate appears in the plasma of volunteers who consumed LC and OE, while ferulic acid 4-O-glucuronide only appears in volunteers who consumed LC. This result may be due to a higher content of ferulic acid derived from verbascoside compared to that of the parent compounds present in OE, as caffeic acid is easily transformed into ferulic acid by the enzyme caffeate O-methyltransferase.^30^ This is consistent with a study in which it was found that when the concentration of a metabolite is lower, sulfation is more effective, while glucuronidation is much more effective than sulfation at higher concentrations.^50^

**Figure 3:**
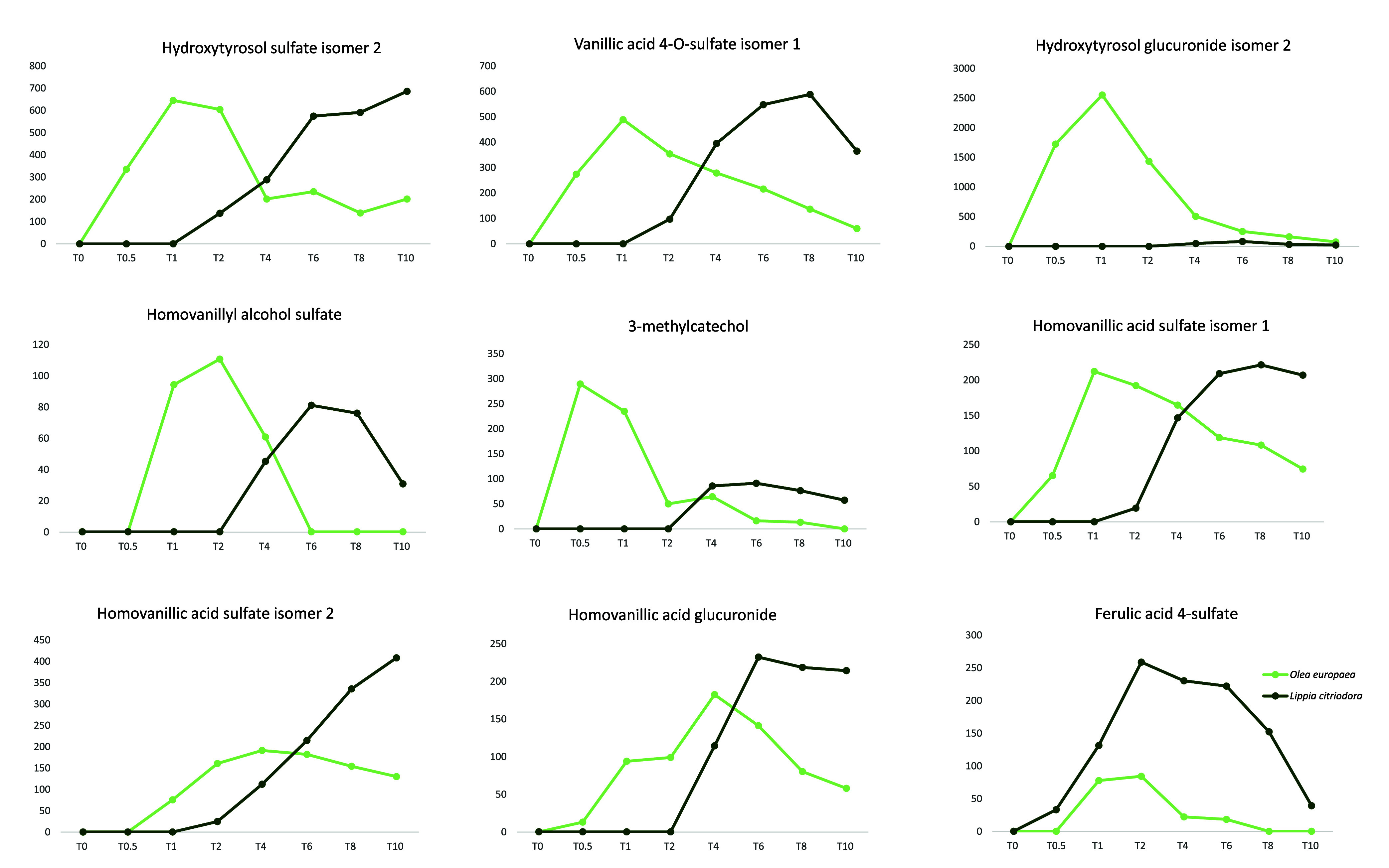
Mean relative abundances of the common annotated metabolites present in plasma between *L. citriodora* and *O. europaea* at different sample collection times. Ordinate axis: relative maximum plasma level (chromatographic area); abscissa axis: hours.

The comparison of the observed *T*_max_ values of the common metabolites reveals that there were statistical differences for this parameter, confirming different metabolization mechanisms between the two matrices (Table 4 and Figure 3)

When examining the AUC graphics of the two common hydroxytyrosol metabolites, hydroxytyrosol sulfate and hydroxytyrosol glucuronide (Figure 3), it becomes apparent that, for OE, the maximum observed *T*_max_ occurs 1 h after ingesting the extract. In contrast, for LC, the AUC values are statistically lower, especially for hydroxytyrosol glucuronide. Furthermore, in the case of the sulfate form, the observed *T*_max_ is 1.1 ± 0.3 h for OE and 8 ± 2 h for LC, while for the glucuronide form, it occurs at 0.9 ± 0.2 and 7 ± 2 h, for OE and LC, respectively. These significant differences might be attributed to the fact that OE extract contains hydroxytyrosol, whereas this compound is not present in LC composition. Therefore, all hydroxytyrosol in LC must first be cleaved from verbascoside. As for hydroxytyrosol glucuronide, the disparity in AUC values and the different times of appearance suggest that hydroxytyrosol, both the free form present in the OE extract and the form resulting from oleuropein cleavage, may undergo phase II metabolic transformations in the liver, as the expression of liver UDP-glucuronosyltransferases is high. In contrast, the hydroxytyrosol present in the LC extract, derived from verbascoside, may be metabolized in the small intestine,^51^ where glucuronidase expression is also present but to a lesser extent, which would explain the lower AUC, since the highest expression occurs in the liver. These findings align with previous results, which indicate that hydroxytyrosol glucuronide in rat plasma results from both phase II metabolism in the liver and the small intestine. Furthermore, the concentration of this metabolite differs based on the source of hydroxytyrosol.^52^

When we focus on the metabolites hydroxytyrosol sulfate, homovanillyl alcohol sulfate, vanillic acid 4-O-sulfate isomer 1, and homovanillic acid sulfate isomer 1 (Figure 3), they exhibit a similar pattern. For the volunteers who ingested LC, there is an observed *T*_max_ at 7 ± 2 h after the compound intake, while those consuming OE have a peak at 2.0 ± 0.9 h, followed by a small resurgence at 8 h. One hypothesis that could explain this is the difference in the locations where sulfation of compounds derived from different matrices occurs. There are studies that indicate a high expression of sulfotransferases in the small intestine,^53^ suggesting that metabolites derived from the OE extract could bind to the sulfate group in the small intestine, explaining their earlier appearance, and later in the liver, as evidenced by a second maximum around 8 h. Meanwhile, LC metabolites could undergo the sulfation reaction in both the small intestine and the liver, and their later appearance could be related to the previous breakdown of verbascoside, undergoing several metabolic reactions before they reach these compounds. These hypotheses are supported by previous studies in which it has been shown that homovanillic acid sulfate has been produced in both the liver and the small intestine.^54,55^ The differences in observed *T*_max_ between the two isomers of homovanillic acid sulfate are notable, suggesting different metabolization mechanisms.^56^

The observed difference in *T*_max_ values for homovanillic acid glucuronide between volunteers who consumed LC and those who ingested OE could be attributed to the metabolic pathway of hydroxytyrosol, from which homovanillic acid is derived. Volunteers who ingested LC may undergo more metabolic reactions before hydroxytyrosol is converted into homovanillic acid glucuronide, leading to a later observed *T*_max_ (9 ± 2 h). In contrast, volunteers consuming OE may experience a more direct metabolic route, resulting in an earlier observed *T*_max_ (4 ± 2 h).

Finally, ferulic acid 4-sulfate is the only compound that, being common in plasma in both matrices, follows the same trend, but with much higher AUC values for LC than for OE, which can be explained by the fact that verbascoside fragments into caffeic acid and is easily transformed into ferulic acid by the enzyme caffeate O-methyltransferase.^30^ If the origin of this metabolite comes from verbascoside, the observed differences in AUC may be related to the fact that the concentration of this compound is much higher in the LC extract compared with that of OE (Table 1).

In conclusion, the study of bioavailable metabolites in plasma from OE and LC extracts using an untargeted approach has shown significant and novel results. This approach has allowed the annotation of 64 circulating metabolites from the OE and LC bioactive extracts, with 9 of them being common between both. The significant differences in observed *T*_max_ for common metabolites suggest the existence of different metabolization mechanisms, which depend on the plant matrix and consequently on the original compounds. This highlights the potential of combining both extracts in the development of nutraceuticals to allow circulating metabolites to reach the bloodstream for a longer period and therefore increase the chances of reaching target tissues.

## Supporting Information Available

Quantification data of the reference standards (Table S1); statistically significant signals related to the intake of the OE and/or LC extracts that could not be annotated (Table S2); mean values of the chromatographic area for each of the times for each compound present in plasma after ingestion of *L. citriodora* extract (Table S3); mean values of the chromatographic area for each of the times for each compound present in plasma after ingestion of *O. europaea* extract (Table S4); 2D PCA scores plots from normalized data including QC samples (Figure S1); 3D PCA scores plots from normalized data including QC samples; MS/MS spectra of vanillic acid sulfate (level 1) (Figure S2); MS/MS spectra of hydroxytyrosol glucuronide (level 1) (Figure S3); and MS/MS spectra of homovanillic acid sulfate (level 1) (Figure S4) (PDF)
