# Supplementary material for: The Application of Untargeted Metabolomic Approaches for the Search of Common Bioavailable Metabolites in Human Plasma Samples from Lippia citriodora and Olea europaea Extracts
Source: J Agric Food Chem. 2024 Oct 22;72(44):24879–93. doi: 10.1021/acs.jafc.4c05325 (PMC11544713; doi:10.1021/acs.jafc.4c05325)
Supplement: Supplementary file 1 — jf4c05325_si_001.pdf [file jf4c05325_si_001.pdf]

Supporting information

The application of untargeted metabolomic approaches for the search of common bioavailable metabolites in human plasma samples from *Lippia citriodora* and *Olea europaea* extracts

María del Carmen Villegas-Aguilar[1], María de la Luz Cádiz-Gurrea[1,*], Noelia Sánchez-Marzo[2], Enrique Barrajón-Catalán[2], David Arráez-Román[1], Álvaro Fernández-Ochoa[1,*,#] and Antonio Segura-Carretero[1,#]

[1]Department of Analytical Chemistry, University of Granada, 18071 Granada, Spain

[2]Institute of Research, Development and Innovation in Biotechnology of Elche (IDiBE) and Molecular and Cell Biology Institute (IBMC), Miguel Hernández University (UMH), 03202 Elche, Spain

Corresponding authors:

Álvaro Fernández-Ochoa, Department of Analytical Chemistry, University of Granada, 18071 Granada, Spain, Tel. +34 958240794, alvaroferochoa@ugr.es

María de la Luz Cádiz-Gurrea, Department of Analytical Chemistry, University of Granada, 18071 Granada, Spain, mluzcadiz@ugr.es

[#] These authors shared author co-senior ship.

Table S1. Standard calibration curves used for the quantification of the compounds of interest in the OE and LC extracts.

**Analytical standard**	**LOD (µM)**	**LOQ (µM)**	**Calibration Range (µM)**	**Calibration Equations**	**R^2**
verbascoside	0.011 ± 0.001	0.033 ± 0.006	(LOQ – 185)	log y = 0.84 log x + 4.48	0.99
oleuropein	0.004 ± 0.002	0.013 ± 0.007	(LOQ – 185)	log y = 0.52 log x + 4.86	0.99
hydroxytyrosol	0.022 ± 0.002	0.052 ± 0.006	(LOQ – 125)	log y = 0.93 log x + 4.13	1

LOD: Limit of Detection; LOQ: Limit of Quantification, R2: Coefficient of variation.

Table S2. Statistically significant signals related to the intake of the OE and/or LC extracts that could not be annotated.

Peak	*L. citriodora**	*O. europaea**	RT (min)	$[M-H]^-$	Molecular formula	MS/MS fragments
1	5	2	1.46	304.9350		-
2	0	5	8.59	414.0648		351.0680, 369.0382
3	0	6	8.91	414.0650		351.0680, 369.0382
4	0	5	8.91	331.0929		-
5	6	0	9.73	436.1082		-
6	5	0	9.73	405.1037	$C_{16}H_{22}O_{12}$	-
7	0	8	10.53	311.0743	$C_{14}H_{16}O_8$	159.8886, 99.9255, 100.9341
8	0	6	10.55	328.0644	$C_{10}H_{17}O_{12}$	-
9	0	5	11.23	281.0644	$C_{20}H_{10}O_2$	-
10	0	6	11.25	344.0596		-
11	6	0	11.8	429.2117		-
12	0	6	12.58	276.0695		-
13	0	5	13.75	328.0648		-
14	8	0	13.76	395.0946	$C_{22}H_{20}O_5S$	-
15	0	8	13.81	311.0741	$C_{14}H_{16}O_8$	-
16	0	7	13.91	311.0741	$C_{14}H_{16}O_8$	-
17	0	8	13.96	571.1668	$C_{29}H_{32}O_{10}S$	-
18	7	0	14.05	429.2110		-
19	0	5	14.10	567.1715		197.0811, 175.0202, 113.0237
20	0	6	14.25	543.1714	$C_{24}H_{32}O_{14}$	-
21	0	5	14.49	459.0596		-
22	0	7	14.62	514.1370	$C_{15}H_{31}O_{19}$	-
23	7	4	14.95	197.0816		-
24	5	0	14.95	287.0510		-
25	5	0	15.02	427.1969		-
26	8	0	15.15	465.1976		-

27	0	7	15.15	459.0954		-
28	0	6	15.45	591.1681	$C_{32}H_{32}O_9S$	-
29	0	8	15.54	580.2024		-
30	0	8	15.59	484.1276		-
31	8	0	15.70	429.2120		-
32	8	0	15.77	467.2134	$C_{20}H_{36}O_{12}$	113.0232, 144.9160, 222.9614
33	6	0	16.28	253.1059		-
34	8	0	16.59	413.2163		-
35	7	0	16.79	479.1899		-
36	1	7	17.89	487.3420	$C_{30}H_{48}O_{15}$	-
37	6	0	18.16	679.3471		-
38	5	0	19.01	465.2094		-
39	8	0	19.12	361.1999		315.1962, 293.2109, 96.9677
40	0	6	23.72	795.3551		-

*Number of volunteers in whom the metabolite appears; RT: retention time.

Table S3. Mean values of the chromatographic area for each of the times for each compound present in plasma after ingestion of *L. citriodora* extract.

n	RT (min)	[M-H]-	molecular formula	proposed compound	T0	T0.5	T1	T2	T4	T6	T8	T10
5	1.46	304.9350		unknown	0	0	0	38	35	0	56	41
7	6.53	233.0122	$C_8H_{10}O_6S$	hydroxytyrosol sulfate isomer 2	0	0	0	138	289	575	591	686
7	7.25	246.9916	$C_8H_8O_7S$	vanillic acid 4-O-sulfate isomer 1	0	0	0	97	395	548	588	365
4	8.92	329.0875	$C_{14}H_{18}O_9$	hydroxytyrosol glucuronide isomer 2	0	0	0	0	48	81	34	23
4	9.24	247.0279	$C_9H_{12}O_6S$	homovanillyl alcohol sulfate	0	0	0	0	45	81	76	31
6	9.26	123.045	$C_7H_8O_2$	3-methylcatechol	0	0	0	0	86	91	76	58
8	9.69	373.1128	$C_{16}H_{22}O_{10}$	gardoside isomer 1	0	0	85	211	86	27	0	0
5	9.73	436.1082		unknown	0	0	86	238	130	26	0	0
7	9.81	261.0075	$C_9H_{10}O_7S$	homovanillic acid sulfate isomer 1	0	0	0	19	147	209	221	207
5	10.29	261.0071	$C_9H_{10}O_7S$	homovanillic acid sulfate isomer 2	0	0	0	25	112	215	336	408
6	10.54	357.082	$C_{15}H_{18}O_{10}$	homovanillic acid glucuronide	0	0	0	0	114	232	219	214
7	10.85	258.9912	$C_9H_8O_7S$	caffeic acid 4-sulfate isomer 1	0	21	89	280	177	86	67	0
6	11.28	258.9924	$C_9H_8O_7S$	caffeic acid 4-sulfate isomer 2	0	0	67	277	266	189	129	22
7	11.58	273.0049	$C_{10}H_{10}O_7S$	ferulic acid 4-sulfate	0	33	131	259	230	222	152	39
6	11.80	429.2117		unknown	0	0	18	116	98	16	0	0
5	12.37	369.0839	$C_{16}H_{18}O_{10}$	ferulic acid 4-O-glucuronide	0	0	0	66	163	217	177	89
5	12.43	201.113	$C_{10}H_{18}O_4$	sebacic acid Isomer 1	0	22	102	35	0	0	0	0
5	12.55	246.9907	$C_8H_8O_7S$	vanillic acid 4-O-sulfate isomer 2	0	18	102	43	0	0	0	0
6	13.45	201.113	$C_{10}H_{18}O_4$	sebacic acid Isomer 2	0	15	86	86	15	0	0	0
6	13.75	373.1132	$C_{16}H_{22}O_{10}$	gardoside isomer 2	0	123	328	480	284	151	51	15
8	13.76	395.0946	$C_{22}H_{20}O_5S$	unknown	0	11	86	133	46	12	0	0
7	14.05	429.211		unknown	0	12	142	25	0	0	0	0
6	14.16	411.2032	$C_{21}H_{32}O_8$	abscisic alcohol 11-glucoside isomer 1	0	50	188	61	0	0	0	0
7	14.95	197.0816		unknown	0	88	236	235	133	72	70	24
5	14.95	287.0510		unknown	0	22	102	84	0	0	0	0
5	15.02	427.1969		unknown	0	21	119	17	0	0	0	0

8	15.15	465.1976		unknown	0	56	192	100	0	0	0	0
8	15.70	429.2120		unknown	0	63	254	206	35	0	0	0
8	15.77	467.2134		unknown	0	112	253	64	0	0	0	0
6	16.12	343.1388	$C_{16}H_{24}O_8$	dihydroconiferin isomer 1	0	44	137	0	0	0	0	0
6	16.28	253.1059		unknown	0	48	141	165	68	47	0	0
6	16.32	291.0863	$C_{15}H_{16}O_6$	picrotoxinin	0	32	102	91	0	0	0	0
5	16.43	343.139	$C_{16}H_{24}O_8$	dihydroconiferin isomer 2	0	34	136	83	0	0	0	0
8	16.59	413.2163		unknown	0	66	170	37	0	0	0	0
8	16.75	411.201	$C_{21}H_{32}O_8$	abscisic alcohol 11-glucoside isomer 2	0	111	519	176	23	0	0	0
7	16.79	479.1899		unknown	0	24	181	12	0	0	0	0
7	17.44	411.2008	$C_{21}H_{32}O_8$	abscisic alcohol 11-glucoside isomer 3	0	56	258	13	13	0	0	0
6	17.92	393.1899	$C_{21}H_{30}O_7$	pteroside Z isomer 1	0	0	105	28	0	0	0	0
8	18.05	393.1909	$C_{21}H_{30}O_7$	pteroside Z isomer 2	0	28	152	97	18	0	0	0
6	18.16	679.3471		unknown	0	0	0	0	0	41	75	66
8	18.38	395.2051	$C_{21}H_{32}O_7$	isopetasoside	0	95	394	200	25	14	0	0
5	19.01	465.2094		unknown	0	0	103	95	48	18	0	17
8	19.12	361.1999		unknown	0	14	154	150	25	0	0	0
8	19.17	293.2116	$C_{18}H_{30}O_3$	17-hydroxylinolenic acid	0	81	428	445	184	31	12	20

n* Number of volunteers in whom the metabolite appears; RT: retention time.

Table S4. Mean values of the chromatographic area for each of the times for each compound present in plasma after ingestion of *O. europaea* extract.

n	RT (min)	[M-H]-	molecular formula	proposed compound	T0	T0.5	T1	T2	T4	T6	T8	T10
6	6.41	233.0124	$C_8H_{10}O_6S$	hydroxytyrosol sulfate isomer 1	0	280	323	178	328	118	202	45
8	6.53	233.0122	$C_8H_{10}O_6S$	hydroxytyrosol sulfate isomer 2	0	336	646	605	202	235	140	202
8	7.25	246.9916	$C_8H_8O_7S$	vanillic acid 4-O-sulfate isomer 1	0	275	489	354	279	216	137	60
5	8.59	414.0648		unknown	0	75	137	21	0	0	0	0
8	8.65	329.0875	$C_{14}H_{18}O_9$	hydroxytyrosol glucuronide isomer 1	0	724	955	463	158	61	28	0
6	8.91	414.065		unknown	0	211	367	193	0	0	0	0
5	8.91	331.0929		unknown	0	38	101	0	0	0	0	0
8	8.92	329.0875	$C_{14}H_{18}O_9$	hydroxytyrosol glucuronide isomer 2	0	1726	2553	1437	507	251	161	75
8	8.93	351.0697	$C_{16}H_{16}O_9$	chlorogenoquinone	0	181	315	154	12	11	0	0
8	9.24	247.0279	$C_9H_{12}O_6S$	homovanillyl alcohol sulfate	0	0	94	111	61	0	0	0
7	9.26	123.045	$C_7H_8O_2$	3-methylcatechol	0	290	235	50	64	16	13	0
5	9.73	405.1037	$C_{16}H_{22}O_{12}$	unknown	0	0	0	0	99	67	30	22
7	9.75	343.0686	$C_{14}H_{16}O_{10}$	vanillic acid glucuronide	0	0	37	77	63	44	22	0
8	9.81	261.0075	$C_9H_{10}O_7S$	homovanillic acid sulfate isomer 1	0	65	212	192	165	119	108	75
8	10.10	343.1033	$C_{15}H_{20}O_9$	homovanillic alcohol glucuronide isomer 1	0	274	503	372	183	66	26	0
8	10.28	259.0819	$C_{11}H_{16}O_7$	3-furanmethanol glucoside isomer 1	0	27	200	184	107	17	0	0
8	10.29	261.0071	$C_9H_{10}O_7S$	homovanillic acid sulfate isomer 2	0	0	76	161	191	182	154	130
8	10.52	181.0867	$C_{10}H_{14}O_3$	1,2,3-trimethoxy-5-methyl benzene	0	117	221	105	0	0	0	0
8	10.53	149.0604	$C_9H_{10}O_2$	p-vinylguaiacol	0	90	184	83	0	0	0	0
8	10.53	311.0743	$C_{14}H_{16}O_8$	unknown	0	129	240	126	13	0	0	0
6	10.54	225.0753	$C_{11}H_{14}O_5$	desoxy elenolic acid	0	56	123	35	0	0	0	0
7	10.54	357.082	$C_{15}H_{18}O_{10}$	homovanillic acid glucuronide	0	13	94	99	182	141	80	58
6	10.55	328.0644	$C_{10}H_{17}O_{12}$	unknown	0	91	161	44	0	0	0	0
8	10.55	243.0870	$C_{11}H_{16}O_6$	threo-syringoylglycerol isomer 1	0	267	442	253	78	0	0	0
6	10.60	211.0606	$C_{10}H_{12}O_5$	eudesmic acid	0	34	120	28	0	0	0	0

7	11.06	411.0904	$C_{22}H_{20}O_6S$	4β-benzylthioepicatechin	0	25	123	151	50	0	0	0
8	11.09	343.1032	$C_{15}H_{20}O_9$	homovanillic alcohol glucuronide isomer 2	0	242	677	704	365	165	61	0
5	11.23	281.0644	$C_{20}H_{10}O_2$	unknown	0	18	94	60	0	0	0	0
8	11.24	259.0819	$C_{11}H_{16}O_7$	3-furanmethanol glucoside isomer 2	0	625	1234	961	483	250	113	0
8	11.24	327.0695	$C_{14}H_{16}O_9$	vanillin glucuronide	0	60	180	121	40	11	0	0
6	11.25	344.0596		unknown	0	96	209	140	35	0	0	0
5	11.58	273.0049	$C_{10}H_{10}O_7S$	ferulic acid 4-sulfate	0	0	78	84	22	18	0	0
8	12.13	229.0714	$C_{12}H_{22}O_4$	decanedioic acid isomer 1	0	0	169	232	167	68	11	0
5	12.40	229.0712	$C_{12}H_{22}O_4$	decanedioic acid isomer 2	0	0	0	26	47	84	38	60
6	12.58	276.0695		unknown	0	111	120	77	32	16	8	0
8	13.09	371.0979	$C_{16}H_{20}O_{10}$	dihydroferulic acid 4-O-glucuronide	0	74	114	94	113	109	74	41
8	13.64	243.0869	$C_{11}H_{16}O_6$	threo-syringoylglycerol isomer 2	0	411	542	309	115	23	14	13
5	13.75	328.0648		unknown	0	56	75	0	0	0	0	0
8	13.81	311.0741	$C_{14}H_{16}O_8$	unknown	0	121	159	67	0	0	0	0
8	13.90	243.0868	$C_{11}H_{16}O_6$	threo-syringoylglycerol isomer 3	0	330	401	233	61	0	0	0
7	13.91	311.0741	$C_{14}H_{16}O_8$	unknown	0	97	132	57	0	0	0	0
8	13.91	555.1712	$C_{25}H_{32}O_{14}$	hydroxyoleuropein isomer 1	0	262	382	181	0	0	0	0
8	13.95	553.1556	$C_{25}H_{30}O_{14}$	oleuropein aglycone glucuronide isomer 1	0	186	202	29	0	0	0	0
8	13.96	571.1668	$C_{29}H_{32}O_{10}S$	unknown	0	151	359	217	23	0	0	0
7	13.97	457.0805	$C_{22}H_{18}O_{11}$	epigallocatechin 7-O-gallate	0	14	126	12	0	0	0	0
5	13.97	593.1486	$C_{27}H_{30}O_{15}$	vicenin-2	0	34	137	46	0	0	0	0
6	14.05	623.1584	$C_{28}H_{32}O_{16}$	Isorhamnetin 3-O-glucoside7-O-rhamnoside	0	34	130	14	0	0	0	0
8	14.05	555.1709	$C_{25}H_{32}O_{14}$	hydroxyoleuropein isomer 2	0	530	841	455	88	0	0	0
5	14.07	585.1815	$C_{26}H_{34}O_{15}$	10-hydroxy-7 -methoxyoleuropein isomer 1	0	0	61	107	16	0	0	0
5	14.10	567.1715		unknown	0	38	94	42	0	0	0	0
5	14.11	541.1562	$C_{27}H_{42}O_{11}$	cortolone-3-glucuronide	0	0	0	0	160	82	63	0

8	14.16	553.1558	$C_{25}H_{30}O_{14}$	oleuropein aglycone glucuronide isomer 2	0	401	482	197	24	0	0	0
6	14.25	543.1714	$C_{24}H_{32}O_{14}$	unknown	0	0	0	0	232	161	154	83
8	14.26	585.1817	$C_{26}H_{34}O_{15}$	10-hydroxy-7 -methoxyoleuropein isomer 2	0	11	164	229	148	34	0	0
6	14.27	569.1868	$C_{26}H_{34}O_{14}$	methoxyoleuropein isomer 1	0	0	92	92	0	0	0	0
5	14.49	459.0596		unknown	0	0	0	18	95	60	60	42
8	14.57	555.1710	$C_{25}H_{32}O_{14}$	hydroxyoleuropein isomer 3	0	0	0	19	113	78	48	0
7	14.62	514.1370	$C_{15}H_{31}O_{19}$	unknown	0	0	147	101	0	0	0	0
8	14.95	555.1712	$C_{25}H_{32}O_{14}$	hydroxyoleuropein isomer 4	0	405	350	71	14	0	0	0
6	15.07	569.187	$C_{26}H_{34}O_{14}$	methoxyoleuropein isomer 2	0	159	123	103	47	25	41	0
7	15.15	459.0954		unknown	0	115	160	17	0	0	0	0
5	15.28	555.1714	$C_{25}H_{32}O_{14}$	hydroxyoleuropein isomer 5	0	227	265	17	0	0	0	0
6	15.45	591.1681	$C_{32}H_{32}O_{9}S$	unknown	0	23	143	0	0	23	0	0
8	15.49	569.1867	$C_{26}H_{34}O_{14}$	methoxyoleuropein isomer 3	0	457	783	450	81	46	0	0
8	15.54	580.2024		unknown	0	192	340	74	42	0	0	0
7	15.56	364.9974	$C_{15}H_{10}O_{9}S$	kaempferol-sulfate	0	25	165	59	0	0	0	0
8	15.59	484.1276		unknown	0	98	447	141	20	0	0	0
7	15.77	201.1130	$C_{10}H_{18}O_{4}$	sebacic acid isomer 3	0	13	0	33	209	226	178	141
8	15.90	393.1184	$C_{20}H_{26}O_{8}$	10-hydroxyoleuropein aglycone	0	165	142	15	0	0	0	0
7	17.89	487.3420	$C_{30}H_{48}O_{15}$	unknown	0	30	40	141	153	102	38	18
5	22.37	535.3086	$C_{33}H_{44}O_{6}$	dihydrocelastryl diacetate	0	0	38	150	163	81	38	20
6	23.72	795.3551		unknown	0	74	123	0	20	52	0	0

n* Number of volunteers in whom the metabolite appears; RT: retention time.

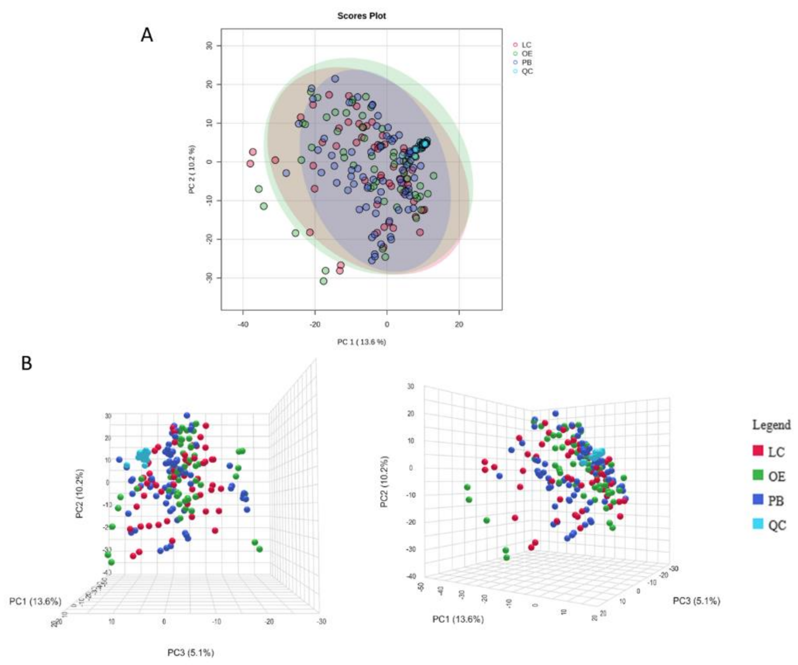

Figure S1. A: 2D PCA scores plots from normalized data for plasma samples. B: 3D PCA scores plots from normalized data for plasma samples.

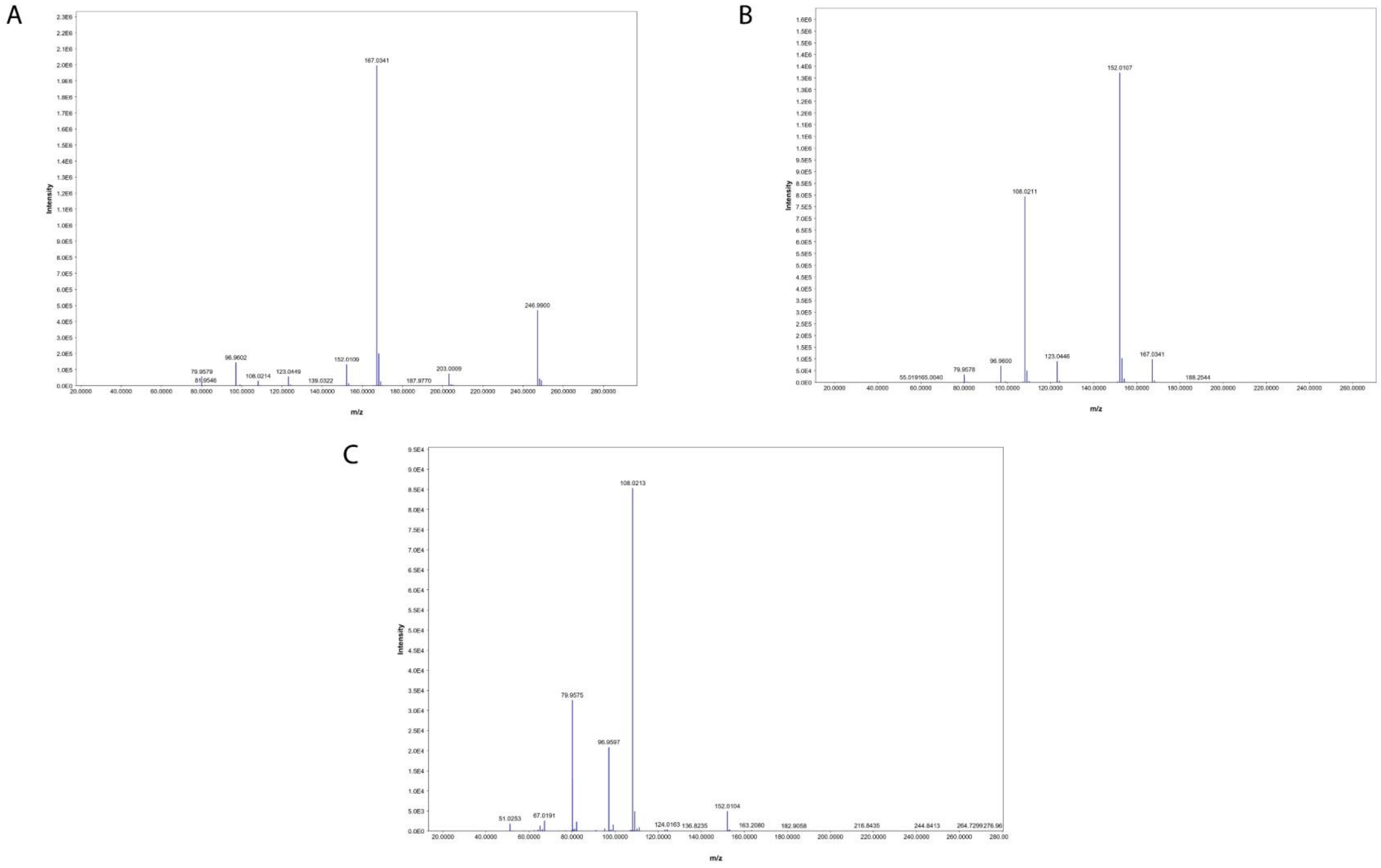

Figure S2. Mass fragmentation spectrum of analytical standard vanillic acid sulfate (m/z: 246.9914). A: collision energies: 10 eV. B: collision energies: 30 eV. C: collision energies: 60 eV.

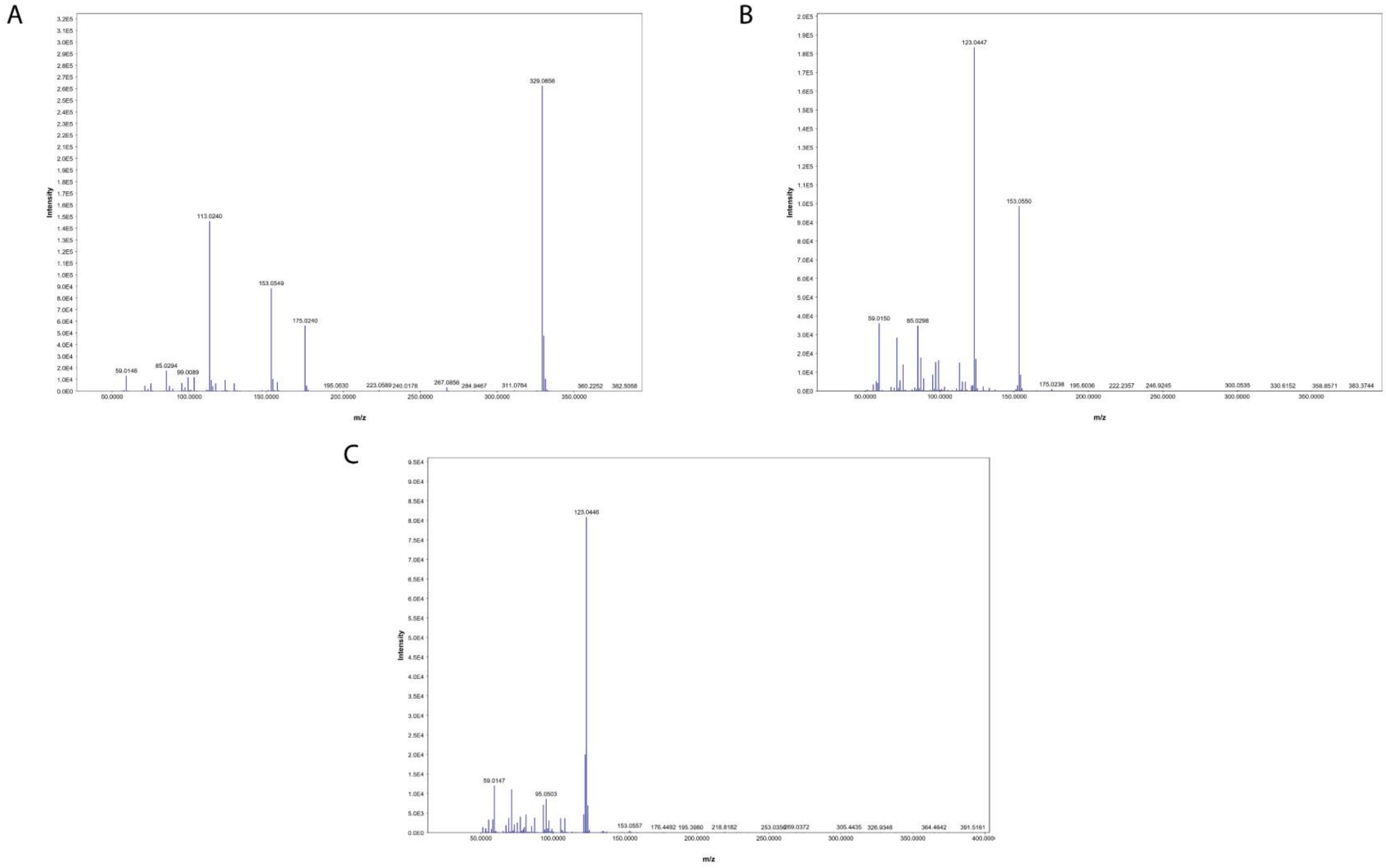

Figure S3. Mass fragmentation spectrum of analytical standard hydroxytyrosol glucuronide (m/z: 329.0856). A: collision energies: 10 eV. B: collision energies: 30 eV. C: collision energies: 60 eV.

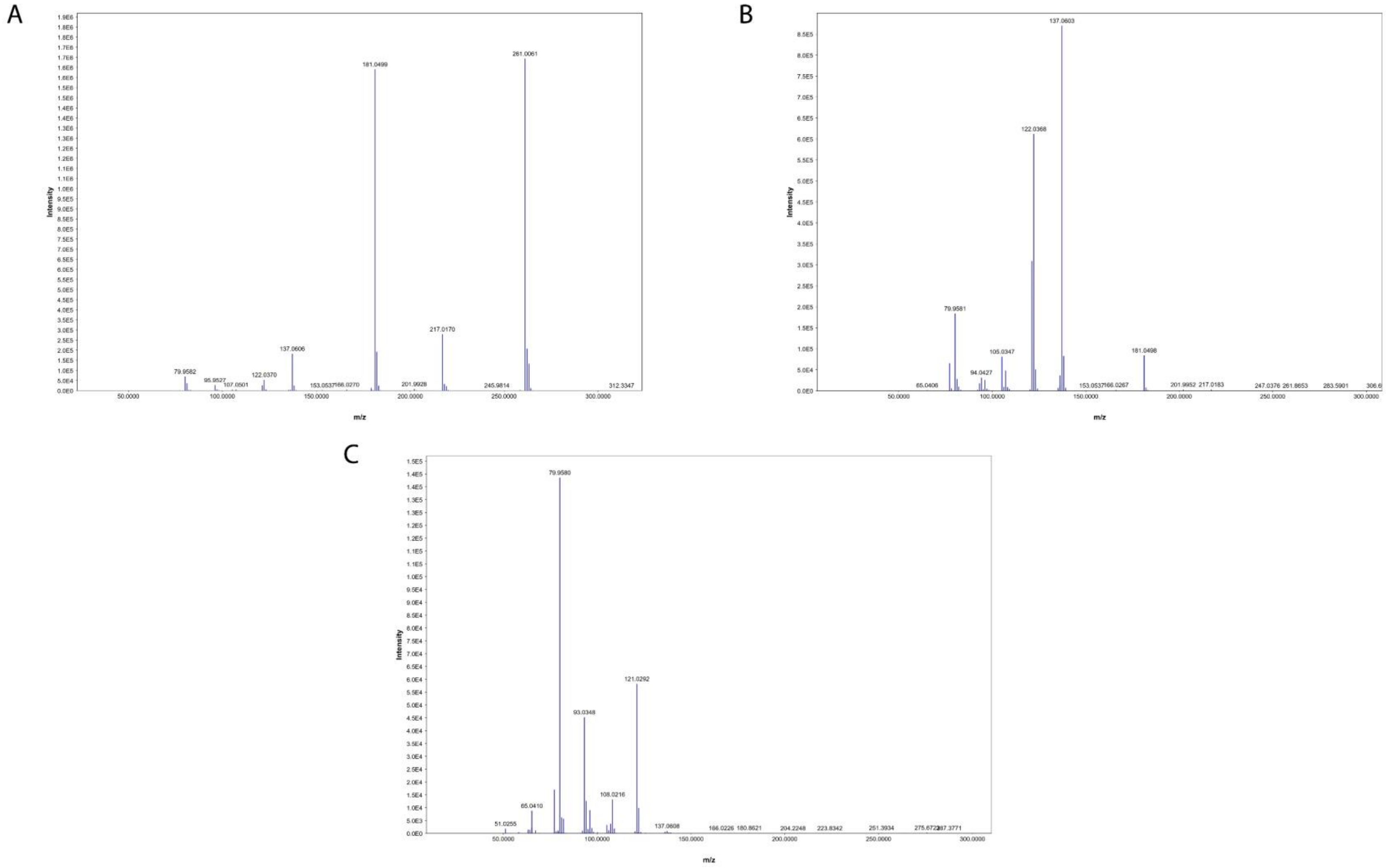

Figure S4. Mass fragmentation spectrum of analytical standard homovanillic acid sulfate (m/z: 261.0061). A: collision energies: 10 eV. B: collision energies: 30 eV. C: collision energies: 60 eV.
